# MiR-206 suppresses the deterioration of intrahepatic cholangiocarcinoma and promotes sensitivity to chemotherapy by inhibiting interactions with stromal CAFs

**DOI:** 10.7150/ijbs.62602

**Published:** 2022-01-01

**Authors:** Renjie Yang, Dong Wang, Shen Han, Yichao Gu, Zhi Li, Lei Deng, Aihong Yin, Yun Gao, Xiangcheng Li, Yue Yu, Xuehao Wang

**Affiliations:** 1 School of Medicine, Southeast University, Nanjing, China.; 2 Hepatobiliary Center, The First Affiliated Hospital of Nanjing Medical University; Key Laboratory of Liver Transplantation, Chinese Academy of Medical Sciences; NHC Key Laboratory of Living Donor Liver Transplantation (Nanjing Medical University), Nanjing, Jiangsu Province, China.

**Keywords:** intrahepatic cholangiocarcinoma, cancer-associated fibroblasts, miR-206, cytokines, exosome

## Abstract

**Background:** Intrahepatic cholangiocarcinoma (iCCA) is a highly malignant subtype of cholangiocarcinoma (CCA) with poor prognosis. In iCCA, the interplay between the stroma and tumor cells results in resistance to adjuvant chemotherapy. Increasing evidence indicates that miR-206 participates in tumor progression, but its role in iCCA is still unclear. The aim of this study was to identify dysregulated miR-206 expression in iCCA and to further explore the underlying mechanism.

**Methods:** MiR-206 expression was proven to be downregulated in iCCA tissues by qPCR, and its correlation with clinical characteristics and prognosis was investigated. iCCA-derived cancer-associated fibroblast cells (CAFs) and normal fibroblast cells (NFs) were isolated and identified. MiR-206 was knocked in or down in CAFs and CCA cells, respectively, to explore the role of miR-206, and coculture of these treated CCAs and CAFs was conducted to explore the effects of miR-206 on their mutual promoting effects. Exosomes carrying miR-206 and an orthotopic mouse model were used to determine the inhibitory effects of miR-206 on iCCA deterioration *in vivo*.

**Results:** We confirmed that miR-206 is a suppressor of iCCA. Overexpressing miR-206 in CCA cells inhibited cell proliferation, migration and invasion. When cocultured with CCA cells, NFs downregulated miR-206 expression, and NFs were susceptible to transforming into CAFs. Moreover, CAFs promoted CCA cell malignant behaviors and gemcitabine resistance. Overexpressing miR-206 in CAFs or CCA cells inhibited this mutual promoting effect. Additionally, when delivered by exosomes, miR-206 suppressed tumor deterioration. And combined with gemcitabine, this treatment resulted in a longer survival time.

**Conclusion:** Our study explained that the interaction between CCA cells and CAFs promoted iCCA deterioration. As a suppressive factor, miR-206 inhibited aggressive characteristics and gemcitabine resistance by interfering with this mutual promoting effect. This research elucidated the molecular mechanism underlying the unfavorable chemotherapeutic response of patients with iCCA, which provided a promising target for iCCA treatment.

## Introduction

Intrahepatic cholangiocarcinoma (iCCA), which occurs in the liver, is a rare subtype of cholangiocarcinoma (CCA) 1. The incidence and mortality of iCCA have been sharply increasing in recent decades 2. Surgical resection is an effective treatment for iCCA patients; unfortunately, some patients with early disease are ineligible for surgical resection because of locally advanced disease or metastasis. Gemcitabine-based regimens are currently widely used in iCCA treatment. However, neither gemcitabine monotherapy nor gemcitabine-combined chemotherapy achieves satisfactory responses in unresectable patients 3. This highlights the importance of identifying new therapeutic options for iCCA.

Currently, tumor aggressiveness is no longer thought to be determined by tumor cells alone. The tumor microenvironment (TME) has been proven to affect tumorigenesis and progression 4-6. In the primary tumor mass, tumor cells regulate the local environment, which may in turn promote tumor cell malignant behaviors. In addition, exposure of tumors to various stimuli, such as chemotherapy drugs, triggers several signalling pathways in the TME, leading to tolerance to chemotherapeutic drugs. Histologically, iCCA is characterized by abundant stroma, and the majority of its malignant behaviors are related to fibrotic and microvessel environments 7. In the stroma of iCCA, alpha-SMA^+^ CAFs are the predominant components 8. Several studies have shown that the alpha-SMA^+^ environment advances tumor stage and predicts unfavorable outcomes 9. As a barrier to chemotherapeutic drugs, CAFs support cancer deterioration in response to stimuli received from CCA cells. Reports show that the presence of CAFs is correlated with chemotherapy resistance and poor clinical outcomes in patients with solid cancers, including iCCA 10, 11. This mutual promoting effect suggests that it is difficult to effectively suppress tumor deterioration with traditional chemotherapy drugs that target only cancer cells. Therefore, targeting the complicated tumor environment has been considered a promising treatment strategy 12, 13.

MicroRNAs (miRs), a class of noncoding RNAs, play important roles in cancer development. Previous studies have shown that miRs regulate the biological behaviors of tumor cells. MiRs have also been reported to participate in TME formation and to be related to tumor deterioration 14. It has been reported that mir-206 acted as an anti-oncogene in many human tumors. Previous studies suggested that its decreased level in various tumors led to tumor cell growth, migration, invasion and apoptosis, including lung, liver, gastric, breast, colorectal cancer 15-19. Besides, miR-206 was proven to play roles in suppressing hepatic stellate cells activation and liver fibrosis 20. However, the role of miR-206 in iCCA is unknown. Here, we identified miR-206 as a suppressor factor and further investigated the role of miR-206 in iCCA deterioration and the underlying mechanism.

## Materials and Methods

### Sample collection and NF and CAF isolation

Tissues were obtained from patients who underwent surgical treatment at the First Affiliated Hospital of Nanjing Medical University. This study was approved by the institutional ethics committee of University and was conducted in accordance with the principles of the Declaration of Helsinki. All the samples were pathologically identified as being iCCA samples. Paired NFs and CAFs were isolated from fresh iCCA patient tumor tissues. NFs were isolated from adjacent tissues, which were located at least 5 cm from the tumor margin. Isolated NFs and CAFs were harvested and identified based on the expression of the fibroblastic marker Vimentin by immunostaining, and CAFs expressed higher levels of alpha-SMA and FAP than NFs (Figure S1).

### RNA-Fluorescence *In situ* Hybridization (FISH)

A FISH kit (Roche, Basel, Switzerland) was used to detect the expression of miR-206 in iCCA tissues and cells. The miR-206 probe was purchased from Sigma-Aldrich (St. Louis, MO, USA). Fluorescence images were captured using a laser confocal scanning microscope (FV1000; Olympus, Tokyo, Japan).

### Cell culture and co-culture

A human normal intrahepatic bile duct cell line (HiBEC cells), two human CCA cell lines (HUCCT1 and RBE cells), the human umbilical vein endothelial cell line (HUVECs) and the 293T cell line were purchased from American Type Culture Collection (ATCC, Manassas, VA, USA). HiBECs, HUVECs and 293T cells were cultured in DMEM supplemented with 1% penicillin/streptomycin and 10% FBS (medium and supplement from Gibco®, Gaithersburg, MD). The HUCCT1 and RBE cell lines were cultured in RPMI-1640 medium supplemented with 1% penicillin/streptomycin and 10% FBS. The NFs and CAFs isolated from human tissues were cultured in Dulbecco's modified Eagle's medium (DMEM)/F12 supplemented 1% penicillin/streptomycin and 15% FBS (medium and supplement from Gibco®, Gaithersburg, MD). Transwell chambers were employed in this study to coculture cells (Figure S2A). Gemcitabine was purchased from MedChemExpress (Shanghai, China). The IC50 values of gemcitabine in the HUCCT1 and RBE cells were detected by CCK-8 assay (gemcitabine IC50 values: 0.0236 µmol/L in HUCCT1 and 2.51 µmol/L in RBE cells, Figure S2B). Cytokines, including IL-1beta, IL-6, IL-8, VEGF-alpha, CXCL12, and TGF-beta1, were purchased from Beyotime Biotechnology (Shanghai, China).

### Exosome isolation, identification, tracing and transfection

Cells were cultured in DMEM/F12 (Gibco, Germany) without FBS for 48 hours in preparation for exosome isolation. Conditioned medium (CM) was collected from the cells. The CM samples were centrifuged at 300 × g for 10 min to remove the dead cells, then centrifuged at 3,000 × g for 10 min to remove the residual debris, and then filtered through a 0.22-µm filter (Nalgene™, Waltham, MA). ExoQuick-TCExosome Precipitation Solution (System Biosciences, Mountain View, CA, USA) was used to extract the exosomes. Transmission electron microscopy (TEM) was used to observe exosome morphology, and nanoparticle tracking analysis (NTA) (Zetasizer Nano ZS90, Malvern Panalytical) was used to determine the size and concentration of the exosomes. CD9, CD63 and Tsg101 expression was analyzed by Western blotting to characterize the isolated exosomes. CM-Dil solution (Invitrogen, Carlsbad, CA, USA) was used to trace the exosomes. The exosomes were loaded with miR-206-mimic using an Exo-Fect Exosome Transfection Kit (System Biosciences). The exosomes carrying the miR-206-mimic were named miR-206-mimic/exo, and those carrying the negative control were named NC-mimic/exo. For the *in vitro* experiments, exosomes were used to treat cells at a concentration of 50 µg protein/10^5^ cells.

### Cell transfection

Negative control (NC) mimic, miR-206-mimic, NC-inhibitor and miR-206-inhibitor were purchased from GeneChem (Shanghai, China). HUCCT1 and RBE cells were transfected with the NC-mimic and miR-206-mimic with Lipofectamine 3000 reagent (Invitrogen, Carlsbad, CA, USA). The NFs isolated from human tissues were transfected with the NC-inhibitor or miR-206 inhibitor. Cy3 labelling was used to trace miR-206. Paired CAFs were transfected with the NC-mimic or miR-206-mimic. LASP1 and Anxa2 overexpression plasmid vectors were synthesized by GeneChem (Shanghai, China).

### Quantitative real-time PCR (q-PCR) analysis

RNA was extracted using TRIzol® reagent, and miR-206 cDNA was synthesized with the miRNA 1st Strand cDNA Synthesis Kit (by stem-loop); cDNA were synthesized from mRNA with the HiScript® II Q RT SuperMix for qPCR (Vazyme, Nanjing, China). Q-PCR analysis was used to assess the miRNA and mRNA expression levels using the Thermal Cycler Dice Detection System with AceQ qPCR SYBR Green Master Mix, according to the manufacturer's protocol (High ROX Premixed; Vazyme, Nanjing, China). The sequences of the primers used in this study are listed in Supplemental Table 1.

### Western blotting (WB), immunohistochemistry (IHC) and immunofluorescence (IF) staining

Cells were lysed by incubation on ice for 20 min. Proteins were extracted with a protein extraction kit (Beyotime, Shanghai) and used for WB assays. Tissue samples were incubated with 10% neutral buffer and then embedded in formalin paraffin for IHC. Antibodies against alpha-SMA, FAP, Ki67, E-cadherin, Vimentin, LASP1, Anxa2, p-STAT3, STAT3, Nanog, CD9, CD68, Tsg101, Bcl-2 and Bax were purchased from Cell Signaling Technology (Danvers, MA, USA). An anti-beta-actin antibody was used as the control. For IF staining, cells were seeded on previously prepared coverslips and incubated for 24 hours. After being fixed with 4% paraformaldehyde, the cells were treated with 0.1% Triton-100 and then incubated with 5% goat serum. Then, the cells were stained with primary and secondary antibodies. Primary antibodies against Vimentin, alpha-SMA and FAP were purchased from Cell Signaling Technology (Danvers, MA, USA). The nuclei were stained with 4',6-diamidino-2-phenylindole (DAPI). Then, fluorescence was detected using a Nikon Eclipse 80i microscope.

### Cell counting kit (CCK)-8 and colony formation assays

A CCK-8 assay was used to assess cell viability. Cells were plated in 96-well plates at 2×10^3^ cells/well (four replicates per group). Cell viability was measured with a microplate reader (Bio-Rad, Hercules, CA, USA). For the colony formation assay, 500 cells were seeded per well. The culture medium was renewed twice a week. When we harvested the colonies, the cells were washed three times with PBS and stained with crystal violet solution. The cell colonies were counted with ImageJ software. Each experiment was performed in triplicate.

### Transwell migration assays, invasion assays, wound healing assays

Transwell migration, invasion and wound healing assays were conducted to monitor cell motility and metastasis. For the Transwell migration assays, 3000 cells (HUCCT1 cells, RBE cells, NFs or CAFs) were added to the upper chamber (Corning, Bedford, MA, USA) in 500 μL serum-free medium. Membranes coated with Matrigel (BD Biosciences, Bedford, MA, USA) were used in the invasion assays. Serum-free medium containing exosomes was added to the upper chamber. The cells were incubated for 24 h at 37 °C in a 5% CO_2_ atmosphere. The cells were fixed with methanol, stained with 1% toluidine blue, and air-dried. The cell numbers were counted using Image-Pro Plus software (IPP; Media Cybernetics Corporation, USA). For the wound healing assays, cells were seeded in six-well plates at 90% confluence. A vertical wound was generated by dragging a plastic pipette tip across the cell monolayer, and the detached cells were removed. The cells were incubated at 37 °C, and phase contrast images of the wounds were captured after 48 hours of incubation. Images of the wound in each sample were captured in at least three fields of vision.

### Sphere formation assay

HUCCT1 and RBE cells were plated in 6-well ultralow attachment plates (Corning Inc., New York, NY, USA) at a density of 100 cells/well. The cells were then cultured for 7 days in serum-free DMEM/F12 medium supplemented with 20 ng/mL epidermal growth factor (EGF, PeproTech, USA), 10 ng/mL basic fibroblast growth factor (bFGF, PeproTech, USA), and 2% B27 supplement (Gibco, Germany). The culture media was supplemented with an additional 2% B27, bFGF and EGF every other day. The colonies were counted and imaged under a light microscope.

### Dual-luciferase reporter assay

PmirGLO reporter plasmids carrying WT or mutant sequences from the 3′UTR of Anxa2 or LASP1 and a miR-206 mimic or scramble control were cotransfected into 293T cells with Lipofectamine 3000 (Invitrogen; Thermo Fisher Scientific, Inc.). After 48 hours, the cells were harvested and lysed. Then, the firefly and Renilla luciferase activities were measured using the Dual-Luciferase® Reporter Assay System (E1910, Promega, USA) according to the manufacturer's protocol.

### Collagen contraction assay

A total of 2 × 10^5^ NFs or CAFs were embedded in a gel composed of rat tail Col-I. The mixture (500 μL) was aliquoted into individual wells of a 24-well plate, and the plate was incubated at 37 °C. After 30 mins, a pipette tip was used to detach the collagen gels from the wells. Then, complete DMEM was added to the wells. Two days later, the collagen gels were imaged, and their diameters were measured.

### Matrigel tube formation assay

HUVECs were diluted in serum-free RPMI 1640 medium, and 4×10^4^ cells in 100 µl were added to each well of a 96-well culture plate that had been precoated with Matrigel (BD Biosciences, Bedford, MA, USA). In addition, 100 µL CM from NC-mimic/CAFs or miR-206-mimic/CAFs was added to the wells (1:1 ratio of CM to serum-free RPMI 1640). Then, the plate was incubated at 37 °C for 8 hours. Tube formation was visualized under an inverted microscope, and the results were analyzed by Image-Pro Plus software (IPP; Media Cybernetics Corporation, USA). The tube structures from three randomly chosen fields of view were imaged under a microscope and analyzed.

### Enzyme-Linked Immunosorbent Assay (ELISA)

Cells were incubated in serum-free medium for 2 days. The concentrations of IL-1beta, IL-6, IL-8, VEGF-alpha, CXCL12 and TGF-beta1 in the culture medium were detected using ELISA kits (R&D, Minneapolis, MN, USA).

### Flow cytometry (FCM)

FCM was performed using a flow cytometer (BD Biosciences, Franklin Lakes, NJ, US). For cell cycle analysis, cells were washed with cold PBS, resuspended in 0.5 mL of 70% alcohol and incubated at 4 °C overnight. The cells were centrifuged and washed with PBS, and 200 µL of cell cycle reagent was added. The cells were incubated for 30 min in the dark at room temperature and analyzed with a cell cycle analysis kit (Multi Sciences, Brentwood, UK). For cell apoptosis analysis, an Annexin V-FITC/PI apoptosis kit (Multi Sciences, Hangzhou) was used to detect apoptotic cells. The cells were fixed with 70% ethanol and incubated at -20 °C overnight before analysis. Gemcitabine was used to induce apoptosis. For cell stemness analysis, the cells were incubated with a CD44-PE antibody and CD133-APC antibody (BD Biosciences).

### TUNEL staining

Xenograft tumors were collected for sectioning and TUNEL staining. TUNEL staining was performed using the TUNEL Apoptosis Detection kit (R&D Systems, Inc.), according to the manufacturer's protocol. Apoptotic cells were observed and images were captured with a fluorescence microscope; green indicates apoptotic cells, and blue indicates nuclei.

### *In vivo* xenograft experiments

To determine the effects of CAFs on tumor growth, a subcutaneous xenotransplantation tumor model was established in 6-week-old BALB/c nude mice (Charles River, USA). Before tumor cells were injected, we injected isolated CAFs (2×10^6^ CAFs in 0.1 mL PBS) alone. Palpable subcutaneous tumors were not detected in the mice, indicating that CAFs are not primarily tumorigenic. Then, we subcutaneously injected a mixture of tumor cells and CAFs into the flanks of mice to observe tumor formation. A mixture of 1.8×10^6^ HUCCT1 cells and 2×10^5^ CAFs in 0.1 mL PBS was injected. Tumor volume was calculated every week according to the following formula: Volume = 0.5×width^2^×length. After 1-2 weeks, the mice were intraperitoneally injected with gemcitabine (50 mg/kg body weight in DMSO) once a week for a total of 2 weeks. All the mice were killed in the 3-4 week, and tumor masses were harvested for further analysis.

Additionally, to simulate the tumor microenvironment in the liver, we also established an orthotopic liver tumor model in 6-week-old BALB/c nude mice. HUCCT1 cells (1.8×10^6^) and CAFs (2×10^5^) were premixed with Matrigel and then injected into a liver lobe with a 27G insulin syringe (Myjector 1 mL, Terumo Corporation, Somerset, NJ). The mice were monitored after injection. When a tumor mass could be observed in the mouse liver lobes and reached at least 1×1 cm (4-5 weeks), exosomes or drugs were administered, and we started to record the survival times of the mice. Gemcitabine (50 mg/kg body weight in DMSO) was administered weekly by intraperitoneal injection. Considering that NFs are not primarily tumorigenic, NF exosomes were collected and stored at -80 °C for miR-206 transfection and *in vivo* injection. MiR-206-modified exosomes (100 µg exosome protein content in 200 µL PBS) were injected into the tail vein. Exosome injections were consecutively administered two times a week. By staining the exosomes with CM-Dil, we confirmed that the exosomes were transmitted to the liver and were absorbed by the liver (Figure S2C). Tumor growth was monitored every 3 days. Survival time was recorded until mice died.

### Statistical analysis

GraphPad Prism 7 software (San Diego, CA, USA) was used to present the results of statistical analysis. All the experiments were performed an average of at least three times. The results are presented as the mean ± SD of each group. Correlations between the clinicopathological parameters and the miR-206 levels were assessed using the chi-square test or Fisher's exact test. The Kaplan-Meier log rank test was used for survival analysis. Univariate and multivariate analyzes were performed with the Cox proportional hazards model in SPSS 21.0. Statistical analysis of *in vitro* experiments was performed with two-tailed Student's t-test. In all cases, a *P* value less than 0.05 was considered statistically significant (* less than 0.05, ** less than 0.01, and *** less than 0.001).

## Results

### MiR-206 was a suppressive factor, and the downregulation of its expression promoted iCCA deterioration

**Figure 1:**
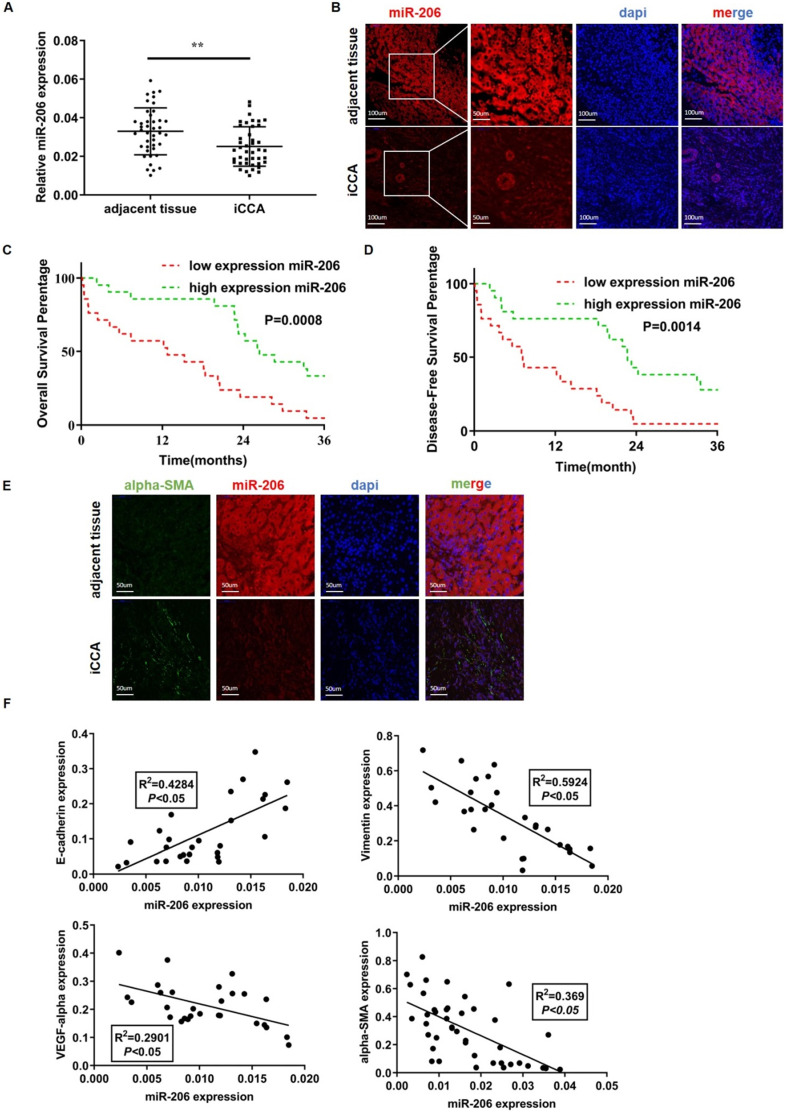
** MiR-206 was a suppressive factor, and the downregulation of its expression promoted iCCA deterioration. (A-B)** Low expression of miR-206 was detected in iCCA by qPCR (n=42) and FISH, scale bars =100 µm and 50 µm. **(C-D)** The overall survival time (n=42, *P*=0.0008) and disease-free survival time (n=42, *p*=0.0014) of patients in the relatively low and high miR-206 expression groups were analyzed and compared. **(E)** Alpha-SMA and miR-206 staining results in iCCA tissues. Scale bar= 50 µm. **(F)** Relationships between miR-206 expression and E-cadherin, Vimentin, VEGF-alpha and alpha-SMA expression in iCCA tissues were analyzed (n=28). The data were shown as the mean ± SD, ** P <0.01, *** P <0.001.

We analyzed 42 human iCCA tissues by qPCR. Compared with paired normal tissues, tumor tissues exhibited obviously lower miR-206 expression (*P*=0.002, Figure 1A). The FISH results showed decreased miR-206 expression in iCCA tissues compared with adjacent tissues (Figure 1B). The 42 patients were then classified into two groups based on miR-206 expression (Figure S2D), and the correlation of miR-206 expression with patient clinical features is summarized in Table 1. miR-206 expression was negatively correlated with larger tumor size (*P* =0.0122) and positively correlated with vascular invasion (*P* =0.0278). Kaplan-Meier analysis showed that low miR-206 expression predicted unfavorable OS (*P* =0.0008) and DFS (*P* =0.0014) rates compared with those predicted by high miR-206 expression (Figures 1C-D). Furthermore, the multivariate analysis results in Table 2 showed that miR-206 expression (*P*<0.001, HR=0.02) could be an independent prognostic factor for unfavorable prognosis. We further analyzed the effects of the tumor stroma on these characteristics. As shown in Table 3, alpha-SMA expression in tissues was also correlated with vascular invasion (*P* =0.0278) and miR-206 expression (*P*<0.001), and low miR-206 expression in tissues was accompanied by a strong alpha-SMA reaction (Figure 1E). We further analyzed the expression of several molecular markers of malignancy, including E-cadherin, Vimentin, VEGF-alpha and alpha-SMA, in iCCA tissues and found that a decrease in miR-206 expression was significantly correlated with an increase in the expression of these markers (E-cadherin: R^2^=0.4284, *P*<0.05, Vimentin: R^2^=0.5924, *P* <0.05, VEGF-α: R^2^=0.2901, *P* <0.05 and alpha-SMA: R^2^=0.369, *P* <0.05) (Figure 1F); these results indicated that loss of miR-206 expression promoted iCCA malignant behavior and stromal reaction.

### Interaction between CCA cells and CAFs resulted in a further reduction in miR-206 expression

**Figure 2:**
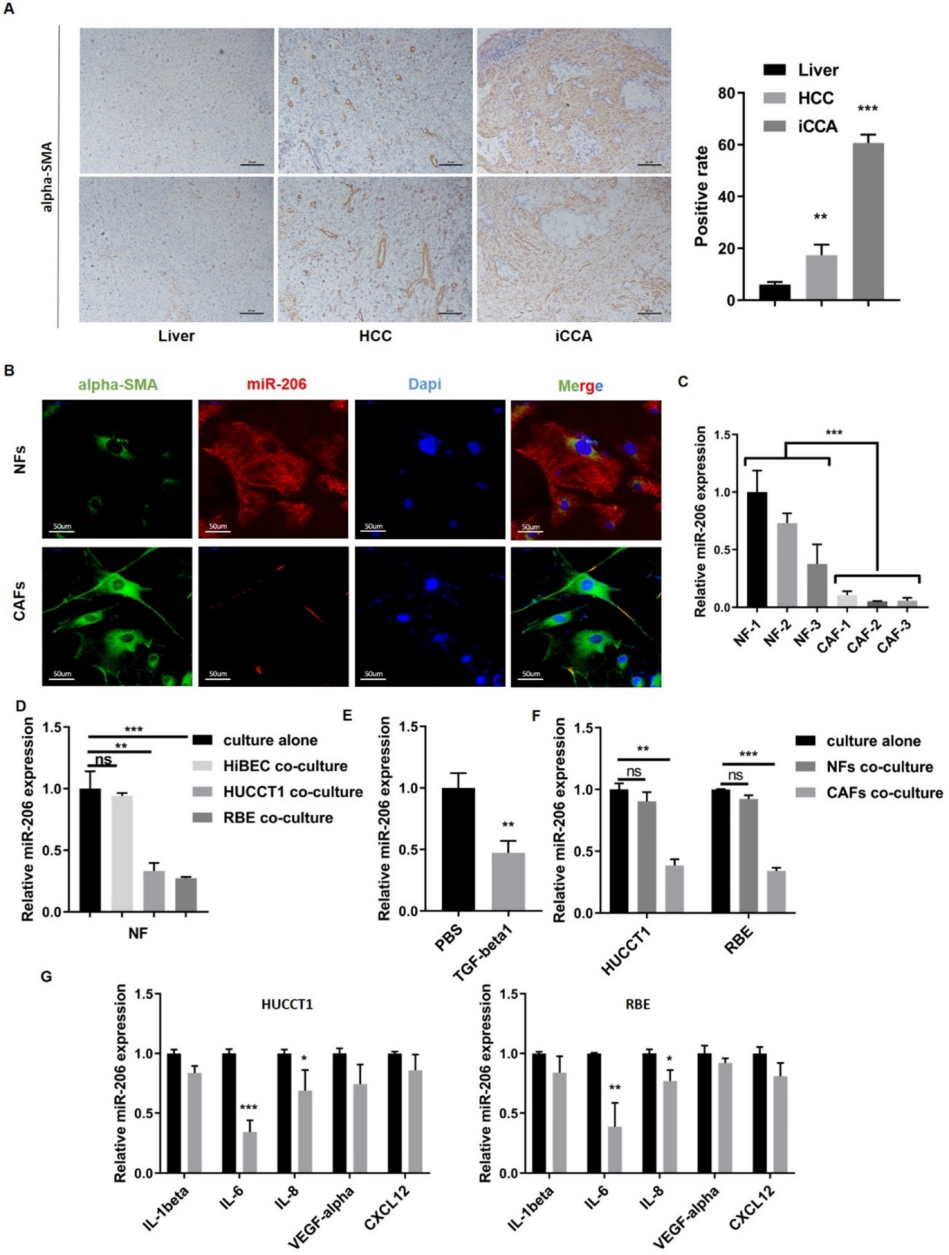
** Interaction between CCA cells and CAFs resulted in a further reduction in miR-206 expression. (A)** Alpha-SMA staining in normal liver, hepatocellular carcinoma and intrahepatic cholangiocarcinoma tissues. The positive staining area was analyzed and is presented in the right panel. Scale bar= 20 µm. **(B)** Alpha-SMA and miR-206 expression in paired NFs and CAFs was detected by FISH. Scale bar=50 µm. **(C)** Expression of miR-206 in 3 pairs of NFs and CAFs was detected by qPCR. **(D)** Relative expression of miR-206 in NFs was analyzed when the NFs were cocultured with HiBEC, HUCCT1 or RBE cells for 48 hours or cultured alone as a control. **(E)** The fold change in the miR-206 levels in NFs treated with or without TGF-beta1 (20 ng/mL for 24 hours) was analyzed. **(F)** Changes in relative miR-206 expression in HUCCT1 and RBE cells cocultured NFs or CAFs were analyzed. **(G)** After treatment with IL-1beta, IL-6, IL-8, VEGF-alpha, and CXCL12 (20 ng/mL for 2 hours), the change in miR-206 expression in the HUCCT1 and RBE cell lines was analyzed, and PBS treatment was used as a control. The data are shown as the mean ± SD, * P <0.05, ** P <0.01, *** P <0.001.

High numbers of neighboring CAFs are a typical characteristic of iCCA tissue (Figure 2A). We isolated paired NFs and CAFs for the following experiments. As shown by qPCR and IF assays, miR-206 expression was decreased in CAFs compared to paired NFs (*P*=0.0002, Figure 2B-C). After coculture with HUCCT1 or RBE cells, the expression of miR-206 in NFs was downregulated (Figure 2D). TGF-beta1, which is secreted by tumor cells, is a well-known profibrotic cytokine, and highly secreted by CCA cell lines compared to HiBEC cell (Figure S2E). After TGF-beta1 stimulation, miR-206 expression in CAFs was sharply decreased (Figure 2E). Then, we incubated HUCCT1 and RBE cells with CM from NFs and CAFs, and a reduction in miR-206 expression was also observed in CAFs group (Figure 2F). We used several cytokines associated with iCCA malignancy to determine the cause of miR-206 downregulation and found that IL-6 caused the most significant reduction in miR-206 expression in both HUCCT1 and RBE cells (Figure 2G, Figure S2F). These results demonstrated that miR-206 was involved in the CCA-CAF interaction, which was mainly attributed to IL-6 and TGF-beta1 secretion.

### MiR-206 suppressed CCA cell proliferation, migration, and invasion and facilitated sensitivity to chemotherapy

**Figure 3:**
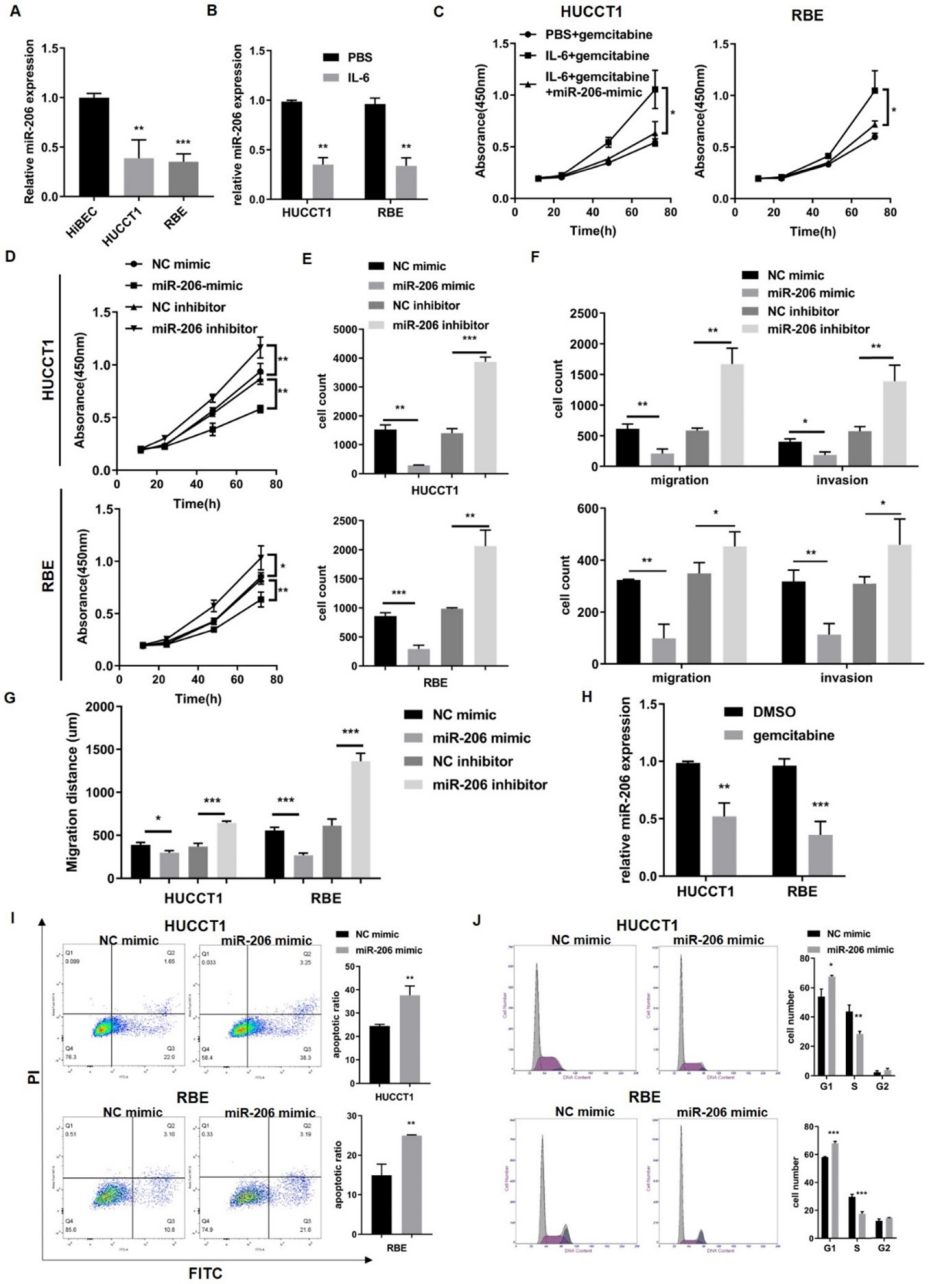
** MiR-206 suppressed CCA cell proliferation, migration, and invasion and facilitated sensitivity to chemotherapy. (A)** MiR-206 levels in two CCA cell lines (the HUCCT1 and RBE cell lines) were analyzed and compared to those in HiBECs. **(B)** MiR-206 expression in HUCCT1 and REB cells was detected after IL-6 treatment. **(C)** The effect of the miR-206 mimic on IL-6-induced HUCCT1 and RBE cell resistance to gemcitabine was detected by CCK-8 assay. **(D-E)** The proliferative potential of HUCCT1 and RBE cells transfected with the miR-206-mimic or miR-206-inhibitor was assessed. CCK-8 and colony formation assay results are presented. **(F-G)** Transwell migration, invasion and wound healing assays were performed to determine the effect of miR-206 on HUCCT1 and RBE cell motility. The Transwell and wound healing assay results are presented. **(H)** The miR-206 levels in HUCCT1 and RBE cells were detected after gemcitabine treatment. **(I)** After treatment with gemcitabine, the apoptosis of HUCCT1 and RBE cells transfected with the miR-206-mimic was detected by FCM. The proportion of apoptotic cells was analyzed and is presented in the right panel. **(J)** Cell cycle distribution of HUCCT1 and RBE cells transfected with the miR-206-mimic was detected. The data are shown as the mean ± SD, * P <0.05, ** P <0.01, *** P <0.001.

To determine the effect of miR-206 on CCA cells, we detected the expression of miR-206 in CCA cell lines (HUCCT1 and RBE cell lines) and found that its expression was downregulated compared to that in HiBECs (Figure 3A). It has been reported that IL-6 contributes to CCA malignancy and chemoresistance. We treated HUCCT1 and RBE cells with IL-6. IL-6 decreased miR-206 expression and enhanced CCA cell resistance to gemcitabine, but this enhanced gemcitabine resistance was attenuated in miR-206-overexpressing cells (Figure 3B-C). This result suggested that miR-206 might be involved in CCA malignant behaviors. We transfected the miR-206-mimic or miR-206-inhibitor into HUCCT1 and RBE cells, and the efficacies of these transfections were confirmed (Figure S3A). The CCK-8 assay showed that overexpression of miR-206 significantly suppressed cell growth and inhibited colony formation in HUCCT1 and RBE cells, while knocking down miR-206 expression promoted cell proliferation (Figure 3D-E, Figure S3B). Transwell migration assays demonstrated that overexpression of miR-206 suppressed HUCCT1 and RBE cell migration, while knocking down miR-206 expression promoted HUCCT1 and RBE cell migration. Invasion assays showed that cells treated with the miR-206 inhibitor were more likely to cross to the other side of the membrane (Figure 3F, Figure S3C), and wound healing assays showed that miR-206 inhibited CCA cell motility (Figure 3G, Figure S3D); all of these results suggested that downregulation of miR-206 expression enhanced cell invasion. Furthermore, we examined the effect of miR-206 on drug resistance. After gemcitabine treatment (0.023 µmol/L for HUCCT1 cells and 2.51 µmol/L for RBE cells) for 2 hours, the qPCR results showed that miR-206 expression was significantly decreased in the cells treated with gemcitabine (Figure 3H). The results of the apoptosis assay showed that in response to gemcitabine, the proportion of apoptotic HUCCT1 and RBE cells transfected with the miR-206 mimic was significantly increased compared with that of CCA cells transfected with the NC-mimic (Figure 3I). Gemcitabine is an anticancer agent that targets the cell cycle. The FCM results showed that miR-206 induced cell cycle arrest in the G1/S phase and inhibited cell cycle progression (Figure 3J).

### MiR-206 suppressed CCA cell stem-like characteristics and TGF-beta1 secretion via LASP1/STAT3 signalling

**Figure 4:**
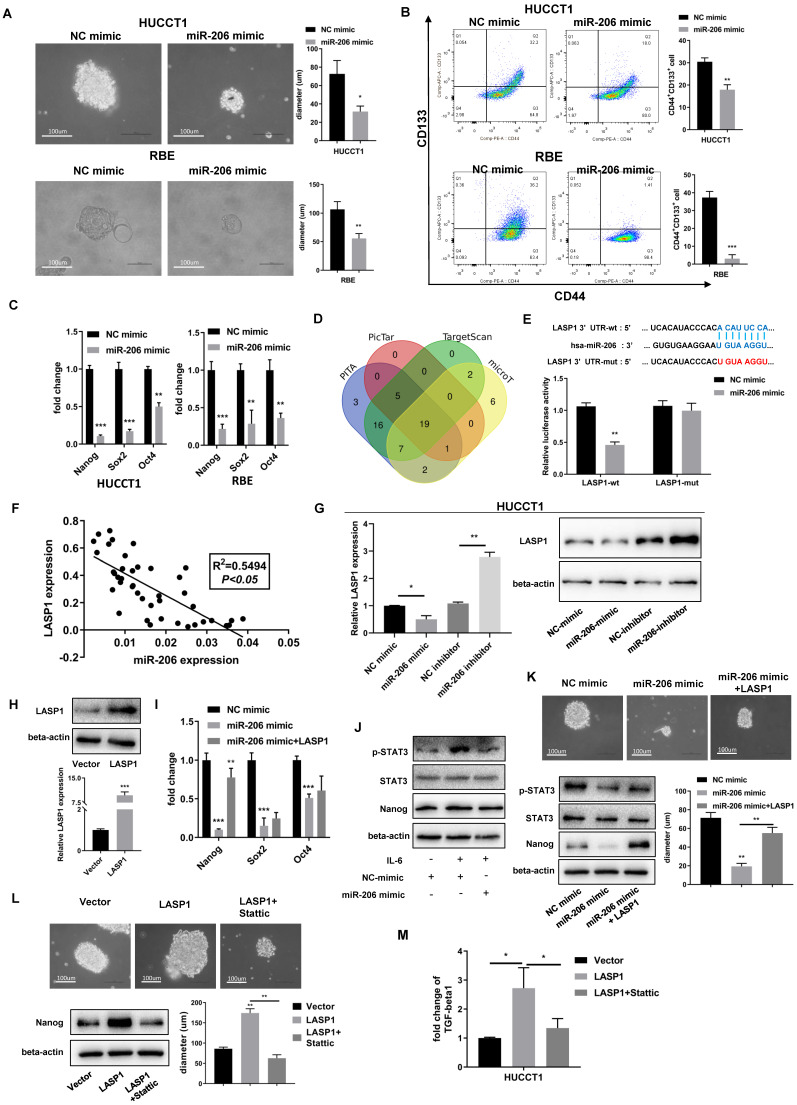
** MiR-206 suppressed CCA cell stem-like characteristics and TGF-beta1 secretion via LASP1/STAT3 signalling. (A)** Representative images of sphere formed by miR-206-mimic/HUCCT1 and miR-206-mimic/RBE cells in serum-free conditioned medium. Colony diameters were analyzed. Scale bar=100 µm. **(B)** The population of CD44+CD133+ cells in the miR-206-mimic/HUCCT1 and miR-206-mimic/RBE cell cultures was detected by FCM. **(C)** mRNA expression of the stem cell regulators Nanog, Sox2, and Oct4 was assessed by qPCR. **(D)** PITA, PicTar, TargetScan and microT were used to predict the target gene of miR-206. **(E)** The binding site of miR-206 in the 3'UTR of LASP1 was predicted, and this binding site was confirmed by a dual-luciferase reporter assay. **(F)** The correlation between miR-206 expression and LASP1 expression in tissues was analyzed (*P*<0.05, R^2^=0.5495). **(G)** Expression of LASP1 in miR-206 overexpression or knockdown HUCCT1 cell line was evaluated by qPCR and WB. **(H)** The overexpression of LASP1 was confirmed by qPCR and WB assays. **(I)** The Nanog, Sox2 and Oct4 mRNA levels in miR-206-mimic/HUCCT1 cells overexpressing LASP1 were analyzed. **(J)** Expression of p-STAT3, STAT3, and Nanog in miR-206-overexpressing HUCCT1 cells after IL-6 treatment. **(K)** Representative images of spheres and expression of p-STAT3, STAT3, and Nanog in the HUCCT1 cell line overexpressing the miR-206 and LASP1. Diameter of sphere was analyzed. Scale bar=100 um. **(L)** Representative images of spheres and protein expression of Nanog in LASP1-overexpressing HUCCT1 cells treated with Stattic (20 μM for 24 hours). Diameter of sphere was analyzed. Scale bar=100 um. **(M)** Secretion of TGF-beta1 by LASP1-overexpressing HUCCT1 cells treated with Stattic (20 μM for 24 hours). The data are shown as the mean ± SD, * P <0.05, ** P <0.01, *** P <0.001.

The stem cell-like characteristics of tumor cells are responsible for drug resistance. The colony formation assay experiments showed that cells overexpressing miR-206 formed more and larger spheroid colonies than those transfected with the NC mimic, and these results were observed both in HUCCT1 and RBE cells (Figure 4A). The results of the FCM assay demonstrated that miR-206 overexpression led to a decrease in the CD44^+^CD133^+^ population compared to the control (Figure 4B). We evaluated the mRNA levels of Nanog, Sox2, Oct4, and regulators of stemness and found that the expression of these regulators was downregulated by miR-206 (Figure 4C).

Through a bioinformatics tool, LASP1 expression was previously reported to correlate with the malignant phenotype of CCA, and LASP1 was predicted to be a direct target of miR-206 (Figure 4D). A predicted binding site in the 3' untranslated region (UTR) of LASP1 mRNA was confirmed by dual-luciferase reporter assay (Figure 4E). Correlation analysis showed that there was a link between miR-206 and LASP1 in tissues (*P*<0.05, R^2^=0.5495, Figure 4F). The results of the WB assay confirmed a decrease in LASP1 expression in miR-206-overexpressing cells and an increase in LASP1 expression in cells treated with the miR-206 inhibitor (Figure 4G). After confirming LASP1 overexpression from a plasmid (Figure 4H), we detected the effect of LASP1 on Nanog, Sox2, and Oct4. The results showed that the downregulation of Nanog expression by miR-206 was significantly reversed by LASP1 overexpression in HUCCT1 cells (Figure 4I). This result demonstrated that LASP1 mediated the inhibitory effect of miR-206 on cell stem-like features by regulating Nanog.

In the IL-6 receptor system, STAT3 signalling is aberrant activated in gemcitabine-resistant cells and correlates with the stem-like phenotype of cells. As shown in Figures 4J-K, miR-206 suppressed STAT3 phosphorylation and Nanog expression, and these effects were mediated by LASP1. Sphere formation assay showed that LASP1 overexpression partially rescued formation of spheroid colonies by HUCCT1 cells. Furthermore, treatment with Stattic, a specific STAT3 inhibitor, suppressed the LASP1-induced sphere formation and expression of Nanog, as well as secretion of TGF-beta1 (Figures 4L-M). These data suggest that LASP1/STAT3 mediated the effect of miR-206 on malignant characteristics of CCA, including drug resistance.

### Reduction of miR-206 expression promoted NF reprogramming toward the CAF phenotype and enhanced IL-6 secretion by targeting Anxa2

**Figure 5:**
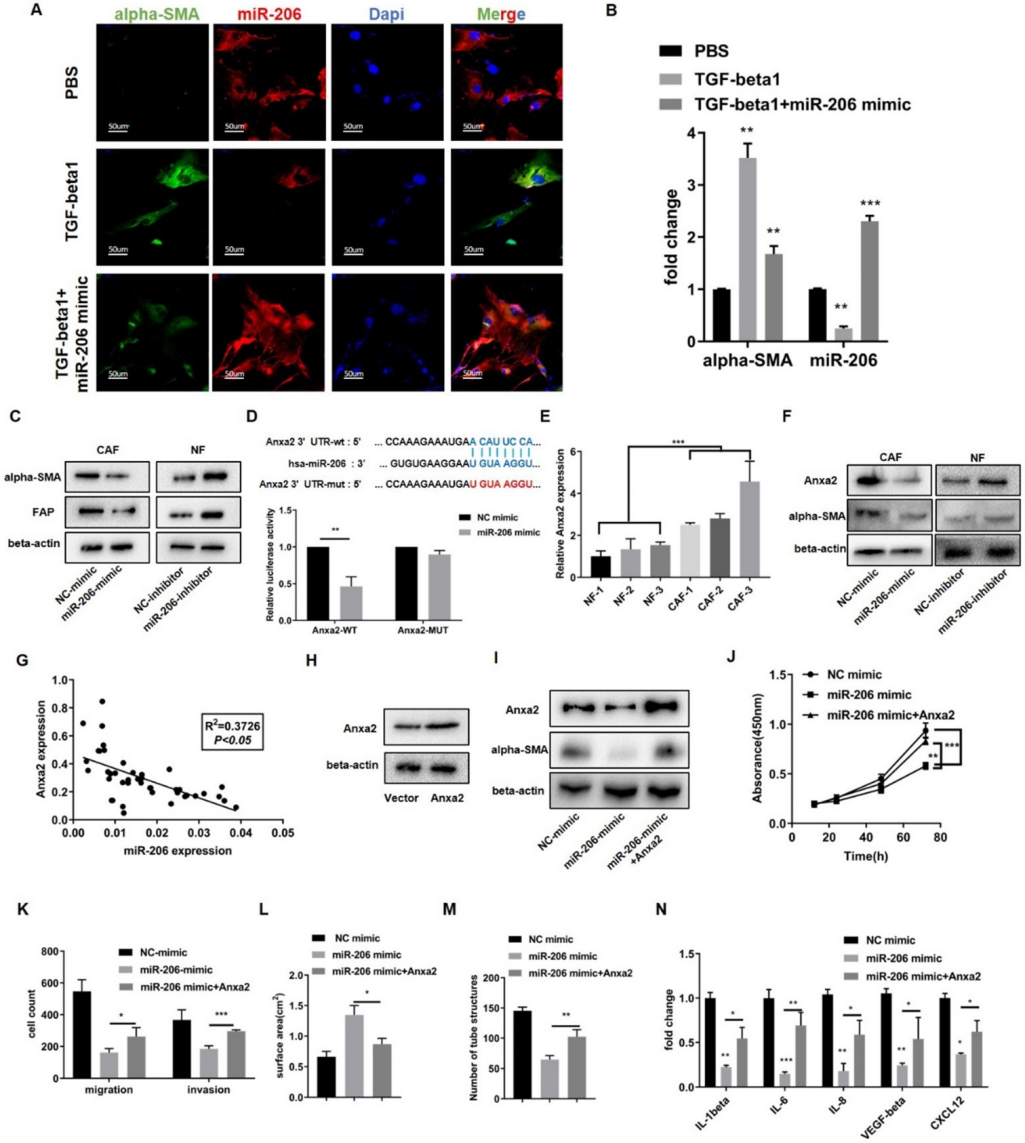
** Decreased miR-206 expression promoted NF reprogramming into the CAF phenotype and enhanced IL-6 secretion by targeting Anxa2. (A)** Expression of alpha-SMA and miR-206 in NFs was evaluated by FISH assay to observe the response of miR-206 expression to TGF-beta1 treatment (20 ng/mL for 12 hours). Scale bar=50 µm. **(B)** Relative changes in the alpha-SMA and miR-206 mRNA levels in response to TGF-beta1 treatment were detected by qPCR. **(C)** The protein expression of CAF markers, alpha-SMA and FAP in miR-206-mimic/CAFs and miR-206-inhibitor/NFs was detected by WB assay. **(D)** The binding site of miR-206 in the Anxa2 3' UTR was predicted, and the binding site was confirmed by dual-luciferase reporter assay. **(E)** Relative expression of Anxa2 in 3 pairs of NFs and CAFs was detected by qPCR. **(F)** Regulation of alpha-SMA and Anxa2 expression by miR-206 was analyzed by WB assay. **(G)** The correlation between miR-206 expression and Anxa2 expression in tissues was analyzed (*P*<0.05, R^2^=0.3726). **(H)** Anxa2 overexpression was confirmed by WB assay. **(I)** Alpha-SMA protein levels were evaluated after Anxa2 expression was upregulated in miR-206-mimic/CAFs. **(J-M)** The effects of Anxa2 in miR-206-mimic/CAFs on CAF proliferation, motility, collagen contraction and vascular formation were studied. **(N)** Fold changes in the IL-1beta, IL-6, IL-8, VEGF-alpha and CXCL12 levels in conditioned medium from miR-206-mimic/CAFs with Anxa2 upregulated expression. The data are shown as the mean ± SD, * P <0.05, ** P <0.01, *** P <0.001.

In response to TGF-beta1, NFs are activated and susceptible to transforming into CAFs, as reflected by the upregulation of alpha-SMA expression. MiR-206 expression was downregulated when NFs were activated, but overexpression of miR-206 sharply decreased alpha-SMA expression (Figures 5A-B). We further studied the effect of miR-206 on CAF activation.

We then transfected CAFs with the miR-206 mimic and NFs with the miR-206 inhibitor. WB results showed that knocking down miR-206 expression in NFs increased the alpha-SMA and FAP levels and promoted NFs activation, while miR-206 overexpression in CAFs decreased alpha-SMA expression (Figure 5C). Subsequent *in vitro* experiments demonstrated that miR-206 inhibited proliferation, migration, pro-collagen contraction, angiogenesis and cytokine secretion (Figures S4A-E). To explain the underlying mechanism, we screened the indicated genes predicted by a bioinformatics tool and found that Anxa2 was a potential target of miR-206 21. A dual-luciferase reporter assay confirmed that Anxa2 was a direct target of miR-206 (Figure 5D). The qPCR and WB results showed that Anxa2 was overexpressed in CAFs compared to paired NFs, and its expression was negatively regulated by miR-206 (Figures 5E-F). Correlation analysis confirmed a link between miR-206 and Anxa2 in tissues (*P*<0.05, R^2^=0.3726, Figure 5G). We transfected CAFs with an Anxa2 overexpression plasmid and confirmed this effect by WB assay (Figure 5H). The results showed that miR-206 decreased the alpha-SMA levels, and this reduction was reversed by Anxa2 (Figure 5I). *In vitro* experiments further demonstrated that Anxa2, which is regulated by miR-206, promoted CAF proliferation, migration, collagen contraction, and angiogenesis (Figures 5J-M, Figures S4F-H). We also analyzed the concentrations of cytokines, including IL-6, in CM from CAFs. The ELISA results showed that Anxa2 reversed the miR-206-mediated suppression of cytokine secretion (Figure 5N).

### Overexpression of miR-206 suppressed the mutual promotion of malignant behaviors and gemcitabine resistance in the CCA-CAF environment

**Figure 6:**
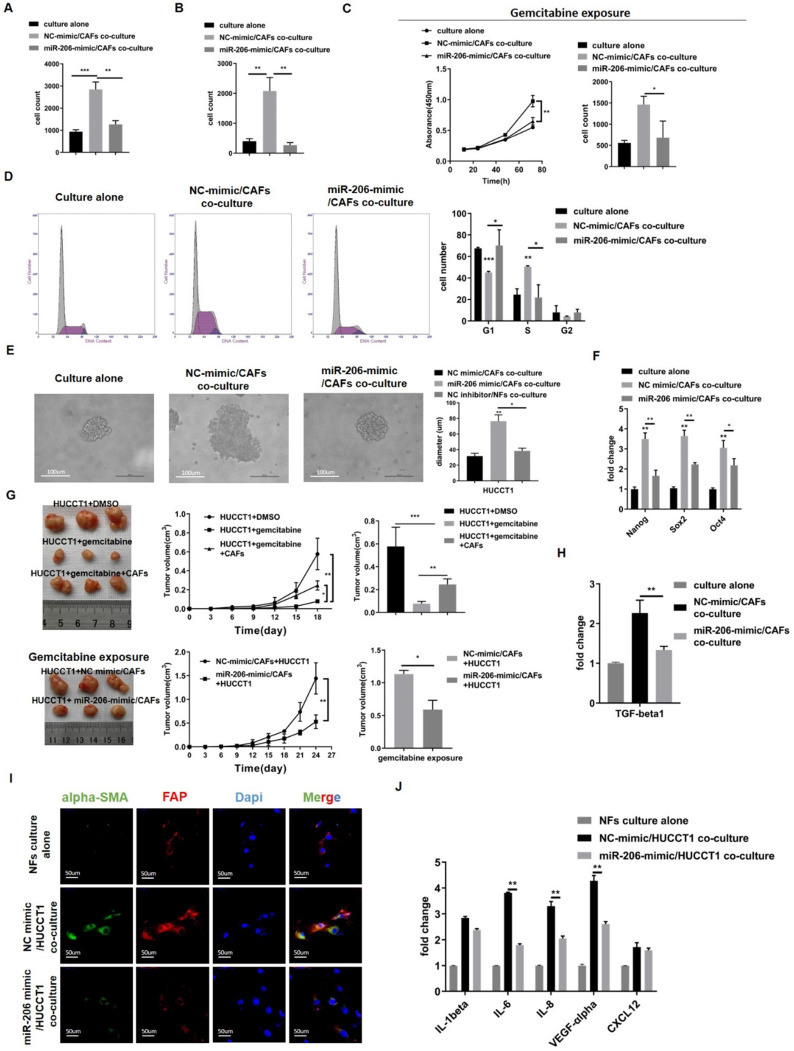
** Overexpression of miR-206 suppressed the mutual promotion of malignant behaviors and gemcitabine resistance in the CCA-CAF environment. (A-B)** The proliferation and migration abilities of HUCCT1 cells cocultured with miR-206-mimic/CAFs were explored by colony formation and Transwell assays. **(C)** The effects of miR-206-mimic/CAFs on HUCCT1 cell resistance to gemcitabine were evaluated by CCK-8 and colony formation assays. **(D)** The cycle distribution of HUCCT1 cells cocultured with miR-206-mimic/CAFs was analyzed by FCM. **(E)** Sphere formation by HUCCT1 cells cocultured with miR-206-mimic/CAFs were detected and assessed. Scale bar=100 µm. **(F)** Expression of Nang, Sox2 and Oct4 in HUCCT1 cells was detected by qPCR. **(G)** MiR-206-mimic/CAFs combined with HUCCT1 cells were subcutaneously coinjected into mice. After gemcitabine treatment for 3-4 weeks, the mice were sacrificed, subcutaneous tumors were imaged, and tumor volume was calculated. **(H)** The mRNA level of TGF-beta1 in HUCCT1 cells cocultured with miR-206-mimic/CAFs was detected by qPCR. **(I)** Alpha-SMA and FAP expression in NFs cocultured with miR-206-mimic/HUCCT1 cells was evaluated by IF assay. NFs culture alone as negative control. Scale bar=100 µm. **(J)** The mRNA levels of IL-1beta, IL-6, IL-8, VEGF-alpha and CXCL12 in NFs cocultured with miR-206-mimic/HUCCT1 cells were detected by qPCR. NFs culture alone as negative control. The data are shown as the mean ± SD, * P <0.05, ** P <0.01, *** P <0.001.

Our results explained the simultaneous reduction in miR-206 expression during CCA-CAF mutual promotion. To explore whether miR-206 overexpression counteracts this mutual promoting effect, we cocultured HUCCT1 cells with CAFs. Colony formation and migration assays showed that HUCCT1 cells cultured with NC-mimic/CAFs exhibited enhanced proliferation and migration potential compared to HUCCT1 cells cultured alone, whereas miR-206-mimic/CAFs exerted a suppressive effect (Figures 6A-B, Figures S5A-B). CCK-8 and colony formation assays showed that in cultures treated with gemcitabine, coculture with NC-mimic/CAFs rescued the growth and colony formation abilities of HUCCT1 cells, and CAFs overexpressing miR-206 (miR-206-mimic/CAFs) significantly suppressed HUCCT1 cell growth (Figure 6C, Figure S5C). Cell cycle analysis showed that CAFs promoted HUCCT1 cell cycle progression from the G1 phase to the S phase, while cell cycle progression was arrested by miR-206-mimic/CAFs (Figure 6D). Sphere formation assay experiments showed that cells cocultured with miR-206-mimic/CAFs formed smaller spheroid colonies than those cocultured with NC mimic/CAFs (Figure 6E). qPCR results showed that Nanog, Sox2 and Oct4 expression in HUCCT1 cells was decreased when they were cocultured with miR-206-mimic/CAFs (Figure 6F). Subcutaneous tumor implantation experiments showed that CAFs coinjection promoted tumor growth when treated with gemcitabine (0.077 cm^3^ vs 0.245 cm^3^, *P*=0.0052), and miR-206 overexpression impaired the effect of CAFs in promoting tumor growth (1.13 cm^3^ vs 0.592 cm^3^, *P*=0.0036, Figure 6G). The level of TGF-beta1 mRNA in HUCCT1 cells was increased when the cells were cocultured with NC-mimic/CAFs, and coculture with miR-206-mimic/CAFs eliminated this upregulated TGF-beta1 mRNA expression (Figure 6H). The results demonstrated that upregulation of miR-206 expression in CAFs attenuated the promotion of malignant behaviors and gemcitabine resistance.

Then, we cocultured NFs with NC-mimic/HUCCT1 cells or miR-206-mimic/HUCCT1 cells and found that NF transformation into CAFs was delayed when cocultured with miR-206-mimic/HUCCT1 cells (Figure 6I). The qPCR results showed that the mRNA levels of IL-1beta, IL-6, IL-8, VEGF-alpha and CXCL12 in CAFs were reduced compared to those in CAFs cocultured with NC-mimic/HUCCT1 cells (Figure 6J). This result suggested that miR-206 was involved in the symbiotic CCA-CAF environment and that the upregulation of its expression may suppress this mutual promoting effect.

### Exosomes acted as carriers of miR-206 in the CCA-CAF environment

**Figure 7:**
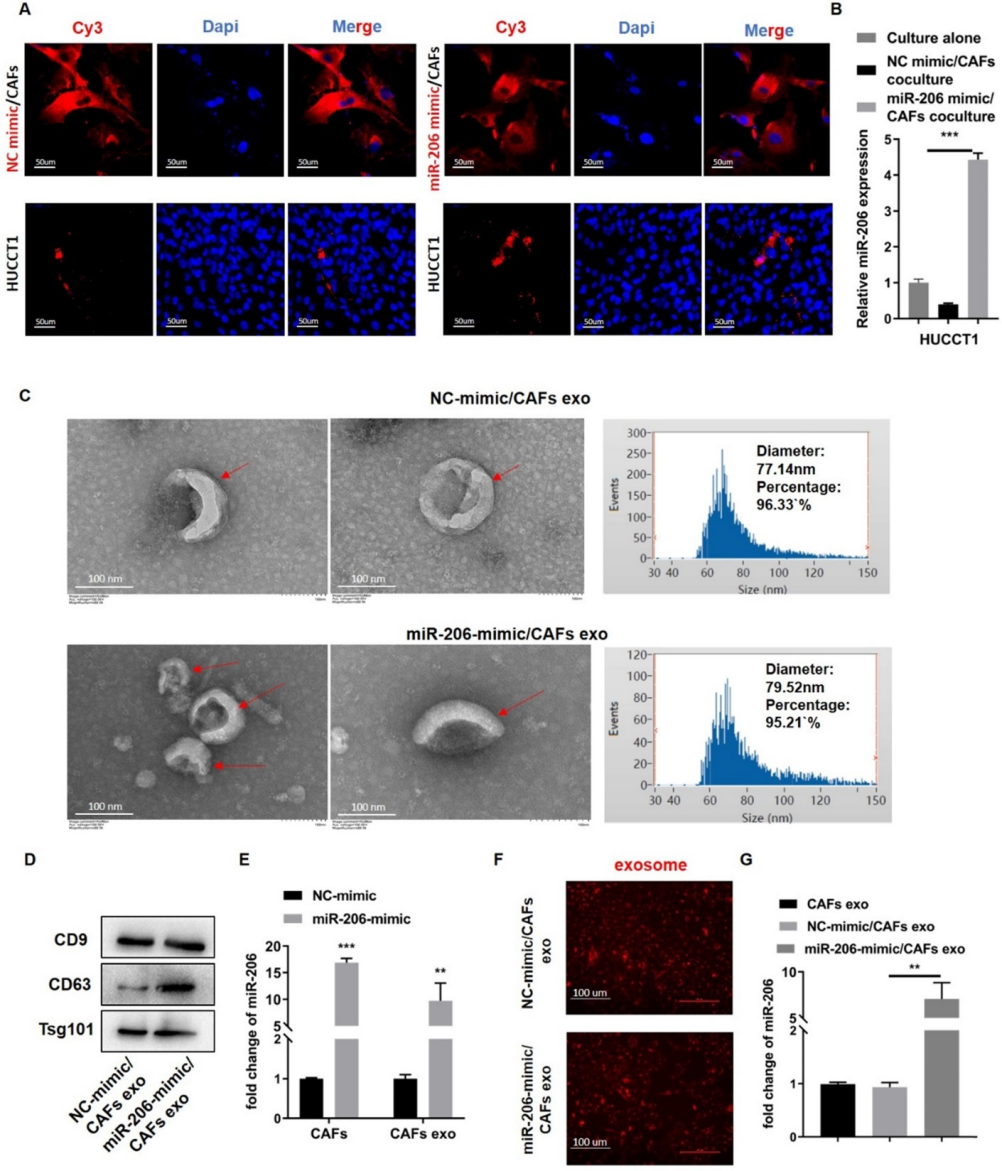
** Exosomes acted as carriers of miR-206 in the CCA-CAF environment. (A)** MiR-206-mimic or NC mimic was labelled with Cy3 and transfected into CAFs. After coculturing these cells with HUCCT1 cells for 48 hours, the labelled NC mimic and miR-206 were observed in the HUCCT1 cells, and representative images were captured. Scale bar=50 µm. **(B)** The level of miR-206 in the cocultured HUCCT1 cells was analyzed by qPCR. **(C)** TEM images of exosomes from NC-mimic/CAFs and miR-206-mimic/CAFs, indicated by red arrows. Scale bar=100 nm. The distribution and size of NC-mimic/CAF- and miR-206-mimic/CAF-derived exosomes were evaluated by NTA. **(D)** WB assay was performed to confirm the protein expression of CD9, CD63, Tsg101. **(E)** Fold changes in miR-206 expression in miR-206-mimic/CAFs and secreted exosomes compared to that in NC-mimic/CAFs and secreted exosomes were analyzed by qPCR. **(F)** NC-mimic/CAF- and miR-206-mimic/CAF-derived exosomes were labelled with CM-Dil (red spot) and cocultured with HUCCT1 cells for 12 hours. The uptake of exosomes by HUCCT1 cells was confirmed by fluorescence microscopy. Scale bar=100 µm. **(G)** The mRNA levels of miR-206 in HUCCT1 cells treated with NC-mimic/CAF-derived exosomes and miR-206-mimic/CAF-derived exosomes relative to those in HUCCT1 cells treated with CAF-derived exosomes were detected by qPCR. The data are shown as the mean ± SD, *P <0.05, ** P <0.01, *** P <0.001.

In coculture systems of miR-206-mimic/CAFs with HUCCT1 cells, Cy3-labelled miR-206 mimic in HUCCT1 cells was observed by fluorescence microscopy, and Cy3-labelled NC mimic was used as the control (Figure 7A). The qPCR results showed that the miR-206 level in the miR-206-mimic/CAF group was much higher than that in the single culture group (Figure 7B). This finding suggests that miR-206 can be transferred during CCA-CAFs communication. We isolated exosomes from CAFs and identified them by TEM, NTA and WB (Figures 7C-D). We analyzed exosomes from miR-206-mimic/CAFs and NC-mimic/CAFs, and we observed an obvious increase in the miR-206 levels in the exosomes from miR-206-mimic/CAFs (Figure 7E). By staining exosomes with CM-Dil, we confirmed that NC-mimic/CAF- and miR-206-mimic/CAF-derived exosomes were internalized by HUCCT1 cells (Figure 7F). HUCCT1 cells treated with exosomes from miR-206-mimic/CAFs displayed an apparent increase in their miR-206 levels (Figure 7G). These observations suggested that exosomes might carry miR-206 and assist in miR-206 translocation into CCA cells.

### Exosome-delivered miR-206 eliminated the CCA-CAF mutually promoting environment and suppressed malignancy

**Figure 8:**
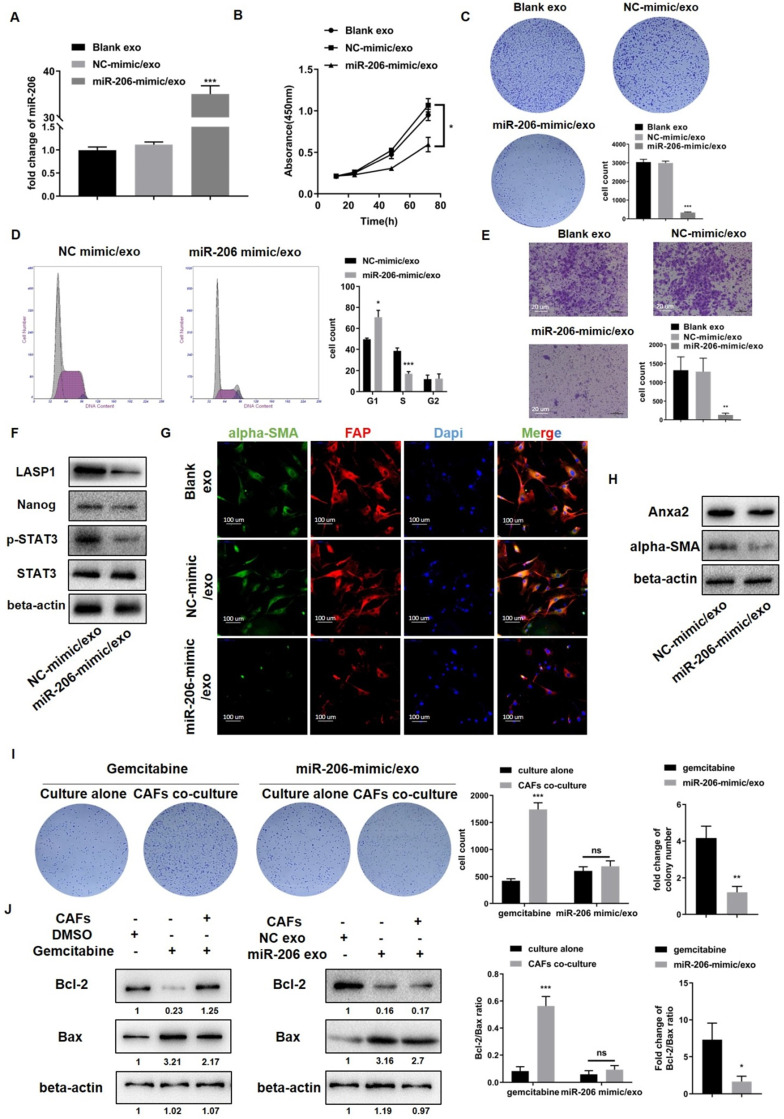
** Exosome-delivered miR-206 eliminated the CCA-CAF mutually promoting environment and suppressed malignancy. (A)** The expression level of miR-206 in -derived exosomes transfected with the miR-206-mimic was detected by qPCR. **(B-E)** Effects of miR-206-mimic/exo (50 μg/10^5^ cells) on HUCCT1 cell proliferation and migration were detected. The CCK-8, colony formation, cell cycle progression and Transwell migration (scale bar=20 μm) assay results are presented. **(F)** Levels of LASP1, Nanog, and p-STAT3 in HUCCT1 cells after miR-206-mimic/exo treatment were assessed. **(G-H)** The expression of CAF markers (alpha-SMA and FAP) and Anxa2 in CAFs was evaluated. **(I)** HUCCT1 cells and CAFs were cocultured in 6-well plates, and both cell types were treated with gemcitabine or miR-206-mimic/exo. The cells were analyzed compared to those cultured alone. Cell colony number and fold change in the gemcitabine and miR-206-mimic/exo treatment groups were compared and are presented in the right panel. **(J)** The Bcl-2/Bax ratio and fold change in the Bcl-2/Bax ratio in the gemcitabine and miR-206-mimic/exo groups were also evaluated and analyzed. *P <0.05, ** P <0.01, *** P <0.001.

To exclude the possible roles of factors other than miR-206 in exosomes, we transfected the miR-206-mimic directly into NF exosomes (miR-206-mimic/exo) and confirmed the success of the transfection (Figure 8A). CCK-8 and colony formation assays showed that compared to NC-mimic/exo, miR-206-mimic/exo significantly inhibited the cell growth and colony formation potential of HUCCT1 cells (Figure 8B-C). Cell cycle analysis demonstrated that miR-206-mimic/exo caused HUCCT1 cell cycle arrest in the G1/S phase (Figure 8D). The migratory behavior of HUCCT1 cells was also suppressed by miR-206-mimic/exo (Figure 8E). WB results showed that miR-206-mimic/exo suppressed LASP1, Nanog, and pSTAT3 expression in HUCCT1 cells (Figure 8F). In CAFs, as shown in IF and WB assays, the expression of CAF markers (alpha-SMA and FAP) and Anxa2 was decreased by miR-206-mimic/exo compared to NC-mimic/exo (Figure 8G-H).

Next, we compared the inhibitory effects of miR-206-mimic/exo with those of gemcitabine. HUCCT1 cells were seeded in 6-well plates, and CAFs were seeded in the upper chamber. Gemcitabine (0.023 µmol/L) or miR-206-mimic/exo (50 µg/10^5^ cells) was added to both chambers, and cell colony formation was observed. In the presence of CAFs, the deleterious effect of gemcitabine was sharply decreased, as shown by the increased colony formation of HUCCT1 cells. In the miR-206-mimic/exo treatment groups, no significant difference in colony formation was observed between the single culture group and coculture group. The fold change in colony number after gemcitabine treatment was much higher than that after miR-206-mimic/exo treatment (Figure 8I). We also detected the expression of the apoptotic markers Bcl-2 and Bax in HUCCT1 cells. The reduction in the Bcl-2/Bax ratio in the gemcitabine group was partially rescued by coculture with CAFs, while this recovery was not observed in the group treated with miR-206-mimic/exo. The fold change in the Bcl-2/Bax ratio after gemcitabine treatment was significantly higher than that after miR-206-mimic/exo treatment (Figure 8J). These results suggested that compared to gemcitabine, miR-206 may eliminate the promoting effects of CAFs and exert stronger inhibitory effects.

### MiR-206-mimic/exo inhibited xenograft malignancy and, when combined with gemcitabine, prolonged the survival time of a mouse model

**Figure 9:**
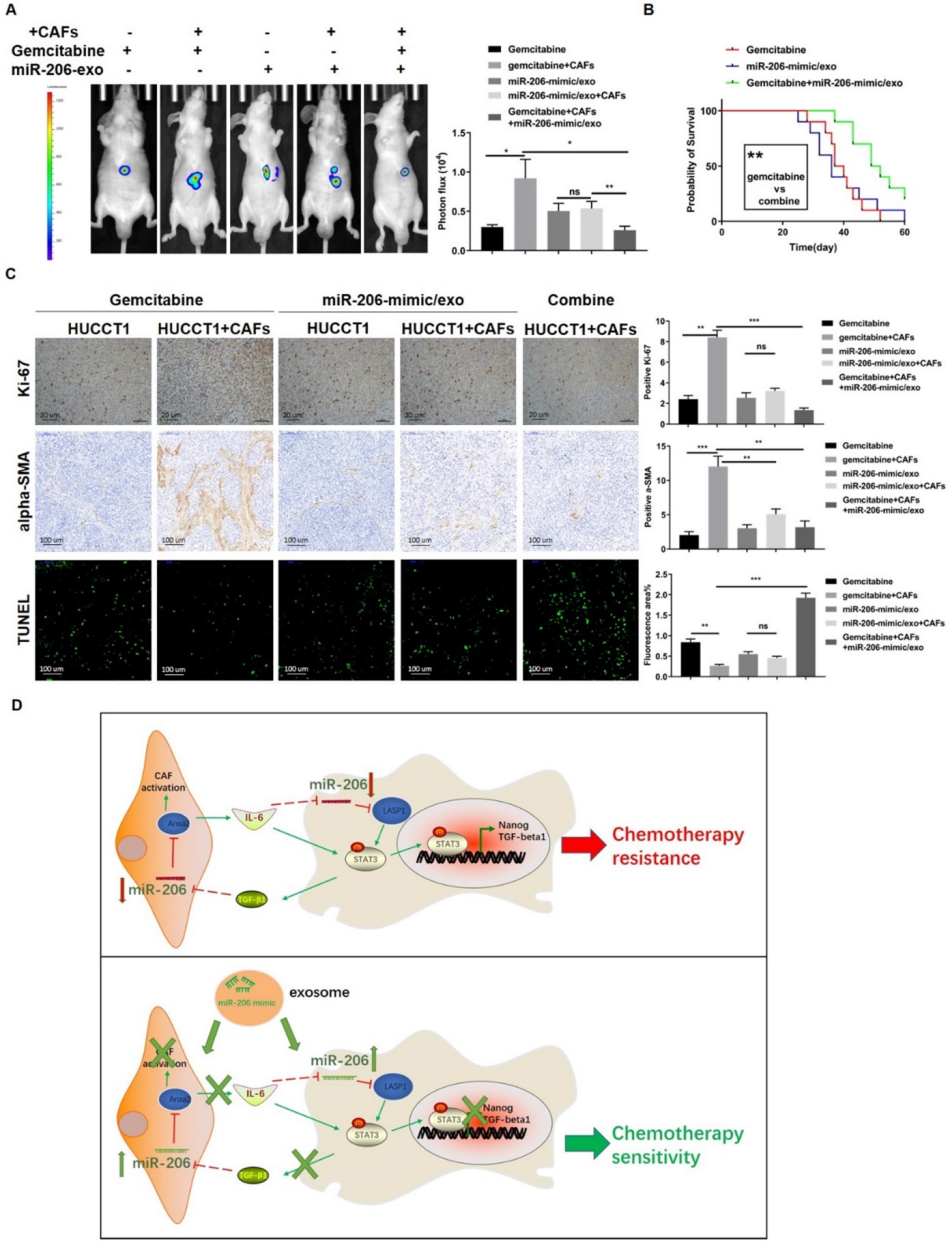
** MiR-206-mimic/exo inhibited xenograft malignancy, and when combined with gemcitabine, prolonged the survival time in a mouse model.** An orthotopic mouse model was established to compare the effects of miR-206 and gemcitabine *in vivo*. Mice were administered miR-206-mimic/exo, gemcitabine or combined treatment. **(A)** After 4 weeks of treatment, the xenograft tumors were photographed (left panel), and the analysis results are shown in the right panel. **(B)** The survival of the mice was monitored, and the survival times of the mice treated with miR-206-mimic/exo, gemcitabine or a combination were analyzed (n=10 in each group). **(C)** After sacrifice, xenograft tumors were harvested and used for IHC staining and TUNEL assays (Scale bars=20 µm for Ki-67 staining, 100 µm for alpha-SMA staining, 100 µm for TUNEL assay). The analysis results were presented in the right panel. **(D)** Schematic representation showing the mechanism by which miR-206 is involved in the CCA-CAF interaction. In CCA cells, miR-206 inactivates the STAT3 pathway and suppresses the expression of Nanog and TGF-beta1. Secreted TGF-beta1 induced the reprogramming of NFs into CAFs via the miR-206/Anxa2 axis and promoted cytokine secretion. Secretion of IL-6 by CAFs further decreased miR-206 expression in CCA cells. miR-206 was involved in the mutual promoting effect that led to iCCA malignancy and resistance to gemcitabine. The administration of exosomes carrying miR-206 can disrupt this mutual promoting effect, and sensitivity to gemcitabine can be increased. *P <0.05, ** P <0.01, *** P <0.001

We further studied the effect of the combination of miR-206-mimic/exo and gemcitabine in an orthotopic mouse model. Tumors were photographed, and the tumor volumes were analyzed. Coinjection with CAFs led to an increase in xenograft volume in the gemcitabine-treated mice, but this increase was not significant in the mice treated with miR-206-mimic/exo. After treatment with gemcitabine combined with miR-206-mimic/exo, the tumor volumes were much smaller than those in mice treated with each reagent alone (Figure 9A). Survival curve analysis showed more favorable outcomes for the mice treated with combined therapy compared with the mice treated with gemcitabine alone (38.5 versus 50.5 days, median survival time, *P*=0.0025) (Figure 9B). As shown by the tissue IHC results, the combined treatment resulted in a lower proportion of Ki-67-positive cells than treatment with each reagent alone (Figure 9C). MiR-206-mimic/exo decreased the alpha-SMA^+^ area in tissues compared to gemcitabine treatment alone (Figure 9C). The TUNEL assay showed that CAFs prevented gemcitabine-induced cell apoptosis, and gemcitabine combined with miR-206-mimic/exo reversed the antiapoptotic effect of CAFs and enhanced the effect of gemcitabine (Figure 9C).

## Discussion

Our results showed that miR-206 was a suppressive factor in iCCA tissues. The downregulation of miR-206 expression was related to the growth and aggressive phenotype of iCCA. Prognosis and multivariate analysis showed that miR-206 was an independent prognostic factor and that decreases in its expression predicted unfavorable patient outcomes.

Tumor is a complex tissue with intricate cell components. Even if tumor cells have the main role, tumor progression cannot be accomplished without other stromal cells, especially in aggressive cancers. Available evidence indicates that CCA microenvironment play a relevant role in its progression and drug resistance. Aggressive phenotype of iCCA is the prototype of tissue containing high amount of CAFs and other extracellular matrix 22. As a result of CAFs' reciprocal communication with CCA cells by paracrine signaling, cancer growth and invasion are driven, and CCA cells acquire cancer stem cell (CSC)-like property and acquired resistance to chemotherapeutic drugs, such as gemcitabine 23. Gemcitabine itself, as a stimulus, induces the iCCA interstitial reaction and increases the expression of IL-6, which enhance tumor cell gemcitabine resistance 24. As a suppressive gene, miR-206 is involved in the interaction between CCA cells and CAFs that promotes the malignancy of CCA cells and enhances their resistance to gemcitabine, and this effect is attributed to IL-6 and TGF-beta1 secretion (Figure 9D).

In cellular experiments, miR-206 inhibited the proliferation, migration and invasion of the HUCCT1 and RBE cell lines. LASP1 (LIM and SH3 domain protein 1) was recently identified as a regulator of tumor progression and drug resistance in several cancers, including CCA 25-28. In this study, LASP1 was proven to be a target of miR-206 and promoted Nanog and TGF-beta1 expression via the STAT3 pathway. TGF-beta1 signalling is aberrantly activated in cancers and plays important roles in the tumor environment, including in the immunity of the tumor environment 29, 30. Additionally, as a profibrotic mediator, TGF-beta1 plays important roles in promoting CAF generation and tumor progression 31-33. A well-known promotor of alpha-SMA expression is Smad3, which is part of TGF-beta1 signaling pathway. Activated TGF-beta1 signaling frequently occurs in stromal component of iCCA 34, 35. After stimulation by TGF-beta1, NFs were susceptible to transforming into CAFs, and this effect was accompanied by a further decrease in miR-206 expression. Previous study proofed that TGF-beta1 inhibited miR-206 expression in airway smooth muscle cell and induced proliferation by activating Smad2/3 36. Blockage of TGF-beta/Smad2 increased miR-206 expression 37. Han* et al.* demonstrated that histone deacetylase 4 (HDAC4) could be induced by TGF-beta1 and recruited to the miR-206 promotor to repress miR-206 transcription during liver fibrogenesis 20. These events suggest that secreted TGF-beta1 acts as an upstream of miR-206 in CAFs and medicated CCA-induced CAFs generation.

In CAFs, we demonstrated that Anxa2 partially mediated the effect of miR-206. It has been reported that Anxa2 (Annexin A2) is a profibrotic gene expressed during tissue fibrosis and cancer 21, 38-40. Mediated by Anxa2, downregulation of miR-206 expression promoted secretion of cytokines, including IL-6, IL-8 and VEGF-alpha. VEGF-alpha, an angiogenesis regulator, is expressed in approximately 50% of iCCA tumors and is associated with increased microvascular density and poor prognosis 41-43. Studies on IL-6 and IL-8 show that they play important roles in protecting pancreatic adenocarcinoma from gemcitabine-induced apoptosis 44, 45. In response to CAFs, miR-206 expression was further downregulated in CCA cells. Our data shows that IL-6 is the main inducer of miR-206 reduction (fall to 0.36-fold approximately, Figure 2G). IL-6 is a multi-functional cytokine, and IL-6/STAT3 signaling activation accounts for 50% of iCCA 46. Influenced by other environmental components and in particular IL-6, STAT3 is activated. Activation of STAT3 plays crucial roles in promoting aggressive phenotype and resistance to a broad spectrum of chemotherapeutic drugs 47. For example, STAT3 activation within triple-negative breast cancer led to epithelial-mesenchymal transition (EMT) phenotype and CSC markers expression, and inhibiting STAT3 enhanced the cisplatin sensitivity 48. Our data show that except for Nanog, miR-206 expression is regulated by IL-6/STAT3 pathway. Previous study has explained that the IL-6/STAT3 pathway might regulate pri-miR-206 posttranscriptionally and reduce mature miR-206 expression 49. These data demonstrate that paracrine IL-6 is main cause of miR-206 reduction in CCA cells.

Moreover, previous studies reported that increased TGF-beta1 and IL-6 contributed to immunosuppressive microenvironment in iCCA. Hasita H *et al.* reported that tumor-associated macrophages (TAMs) contribute to angiogenesis, immunosuppression and poor clinical prognosis via STAT3 activation and TGF-beta1 secretion in iCCA 50, 51. Other types of immune cells, such as NK cells, neutrophil, were also affected by TGF-beta1 52, 53. Through the influence of CCA-CAFs crosstalk, CCA stroma contains an abundance of immunosuppressive immune cells, which facilitate tumor invasion and progression 54.

Current researches indicate that targeting crosstalk between cells populating local environment and CCA cells is a novel promising approach for the management of CCA. A recent study showed that IL-6 was highly expressed in murine and human CCA tissue, and targeting IL-6 signalling inhibited CCA progression *in vitro* and *in vivo* 55. TGF-beta signaling is also an effective target to suppress CCA cell's proliferation and survival 56. By transfecting a miR-206 mimic into either tumor cells or CAFs, IL-6 and TGF-beta1 expression decreased. We observed that the mutual promoting effect between CCA cells and CAFs could be attenuated, and CCA cell malignancy could be inhibited. These results suggest that miR-206 could be used as a drug to inhibit tumor growth and increase gemcitabine efficiency.

Increasing numbers of studies have shown that the development of therapeutic exosomes may lead to decreased immune rejection and improved safety compared to established cellular and drug-based therapeutics 57. As endogenous vesicles, exosomes are refined biological nanoplatforms. Engineering exosomes has been used in cancer therapy as stable transfer of drugs, therapeutic miRNA and proteins 58. In this work, we confirmed that miR-206 mimic could be delivered by fibroblasts-derived exosomes, and this transfer was not observed in CCA cells' exosomes (Figure S6). Following experiments showed exosomes carrying miR-206 exhibited improved delivery efficiency compared to free miR-206 (Figures S7A-C). CCK-8 and qPCR assays showed that NF-derived exosomes carrying miR-206 exerted more significant anticancer effects than CAF-derived exosomes carrying miR-206 (Figures S7D-E). This effect might be attributed to the higher concentration of malignancy-promoting molecules in CAF-derived exosomes than in CAF-derived exosomes 59, 60. Therefore, we used NF-derived exosomes as carriers to observe the anticancer effect of miR-206. As shown in Figure 8, miR-206-mimic/exo eliminated the CCA-CAF mutual promoting effect and improved gemcitabine sensitivity, evidenced by increased TUNEL staining.

In conclusion, our research demonstrates that miR-206 exerts its antitumor effect by inhibiting the malignancy of tumor cells and stromal fibroblasts. MiR-206 promotes iCCA sensitivity to gemcitabine and facilitates gemcitabine-induced tumor cell death mainly by suppressing the mutual promoting effects of tumor cells and stromal fibroblasts. This work elucidated the mechanism underlying drug resistance and highlights the potential of miR-206 as a new therapeutic target.

## Supplementary Material

Supplementary figures and table.

## Figures and Tables

**Table 1:** Correlation between miR-206 expression and clinical features of iCCA patients

Index	iCCA		
	High miR-206 expression	Low miR-206 expression	*P*
**Sex**			
Female	7	6	0.999
Male	14	15	
**Age**			
< 60	14	9	0.2146
≥ 60	7	12	
**Tumor number**			
1	19	17	0.6628
>1	2	4	
**Tumor size**			
< 5	16	7	0.0122*
≥ 5	5	14	
**AFP**			
< 25	19	18	0.999
≥ 25	2	3	
**CA19-9 level**			
< 37	7	5	0.7337
≥ 37	14	16	
**Cirrhosis**			
(-)	18	16	0.6965
(+)	3	5	
**Histology**			
I-II, II	10	7	0.5303
II-III, III	11	14	
**Vascular invasion**			
(-)	16	8	0.0278*
(+)	5	13	

*as* P*<0.05.

**Table 2:** Univariate and multivariate analyzes of miR-206 expression with clinical features of patients

Variable	Univariate analysis			Multivariate analysis		
	*P* value	HR	95 CI%	*P* value	HR	95 CI%
**Sex**						
Female	0.974	1.012	0.495-2.069			
Male						
Age						
< 60	0.275	1.018	0.986-1.051			
≥ 60						
**Tumor number**						
1	0.983	0.99	0.383-2.557			
>1						
**Tumor size**						
< 5	0.089	1.062	0.991-1.139			
≥ 5						
AFP						
< 25	0.85	1	0.999-1.002			
≥ 25						
**CA19-9 level**						
< 37	0.044*	1.001	1-1.002	0.374	1.00	1-1.001
≥ 37						
**Cirrhosis**						
(-)	0.329	0.67	0.3-1.497			
(+)						
**Histology**						
I-II, II	0.408	1.336	0.672-2.665			
II-III, III						
**Vascular invasion**						
(-)	0.002*	2.953	1.482-5.885	0.738	1.141	0.526-2.474
(+)						
**miR-206 expression**						
low	<0.001*	0.014	0.002-0.111	0.001*	0.02	0.002-0.187
high						

*as* P*<0.05.

**Table 3:** Correlation between alpha-SMA expression and clinical features of iCCA patients

Index	iCCA		
	Low alpha-SMA expression	High alpha-SMA expression	*P*
**Sex**			
Female	9	4	0.1809
Male	12	17	
**Age**			
< 60	13	10	0.5359
≥ 60	8	11	
**Tumor number**			
1	19	17	0.6628
>1	2	4	
**Tumor size**			
< 5	15	8	0.0616
≥ 5	6	13	
**AFP**			
< 25	18	19	0.999
≥ 25	3	2	
**CA19-9 level**			
< 37	6	6	0.999
≥ 37	15	15	
**Cirrhosis**			
(-)	17	17	0.999
(+)	4	4	
**Histology**			
I-II, II	10	7	0.5303
II-III, III	11	14	
**Vascular invasion**			
(-)	16	8	0.0278*
(+)	5	13	
**miR-206 expression**			
Low	2	19	<0.001*
High	19	2	

*as* P*<0.05.

## Abbreviations

CCA: cholangiocarcinoma
iCCA: intrahepatic cholangiocarcinoma
miR: microRNA
TME: tumor microenvironment
CAF: cancer-associated fibroblast
NF: normal fibroblast
exo: exosome
alpha-SMA: alpha-smooth muscle actin
FAP: fibroblast activation protein, alpha
FISH: fluorescence *in situ* hybridization
IL: interleukin
VEGF-alpha: vascular endothelial growth factor alpha
CXCL: chemokine
TGF-beta: transforming growth factor beta
WT: wild type
CSC: cancer stem cell
EMT: epithelial-mesenchymal transition
TEM: transmission electron microscopy
NTA: nanoparticle tracking analysis
NC: negative control
qPCR: quantitative real-time PCR
WB: Western blotting
IHC: immunohistochemistry
IF: immunofluorescence
CM: culture medium
ELISA: enzyme-linked immunosorbent assay
FCM: flow cytometry
STAT: signal transducer and activator of transcription
