# MiR-206 suppresses the deterioration of intrahepatic cholangiocarcinoma and promotes sensitivity to chemotherapy by inhibiting interactions with stromal CAFs: Erratum

**DOI:** 10.7150/ijbs.75760

**Published:** 2022-07-08

**Authors:** Renjie Yang, Dong Wang, Shen Han, Yichao Gu, Zhi Li, Lei Deng, Aihong Yin, Yun Gao, Xiangcheng Li, Yue Yu, Xuehao Wang

**Affiliations:** 1 School of Medicine, Southeast University, Nanjing, China.; 2 Hepatobiliary Center, The First Affiliated Hospital of Nanjing Medical University; Key Laboratory of Liver Transplantation, Chinese Academy of Medical Sciences; NHC Key Laboratory of Living Donor Liver Transplantation (Nanjing Medical University), Nanjing, Jiangsu Province, China.

The authors realized three errors in our paper. We regret that we did not detect these errors before publication. The images of figure 4H and figure 6I were inaccurate. The image of figure 9C had been misused during figure assembly. These errors were unintentionally introduced during figure assembly. Here we showed the corrected figure 4H, figure 6I and figure 9C with side by side comparison of the previous figures. These revisions do not alter scientific conclusion in this work.

## Figures and Tables

**Figure 4:**
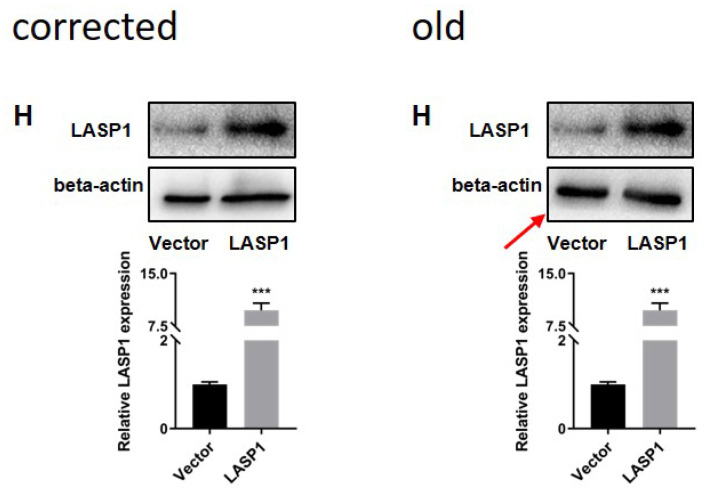
** H.** Corrected figure.

**Figure 6:**
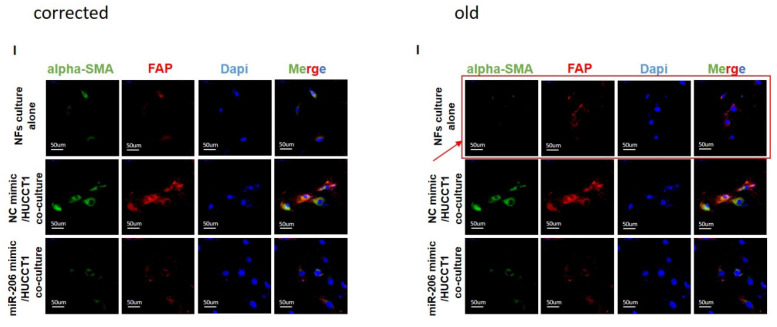
** I.** Corrected figure.

**Figure 9:**
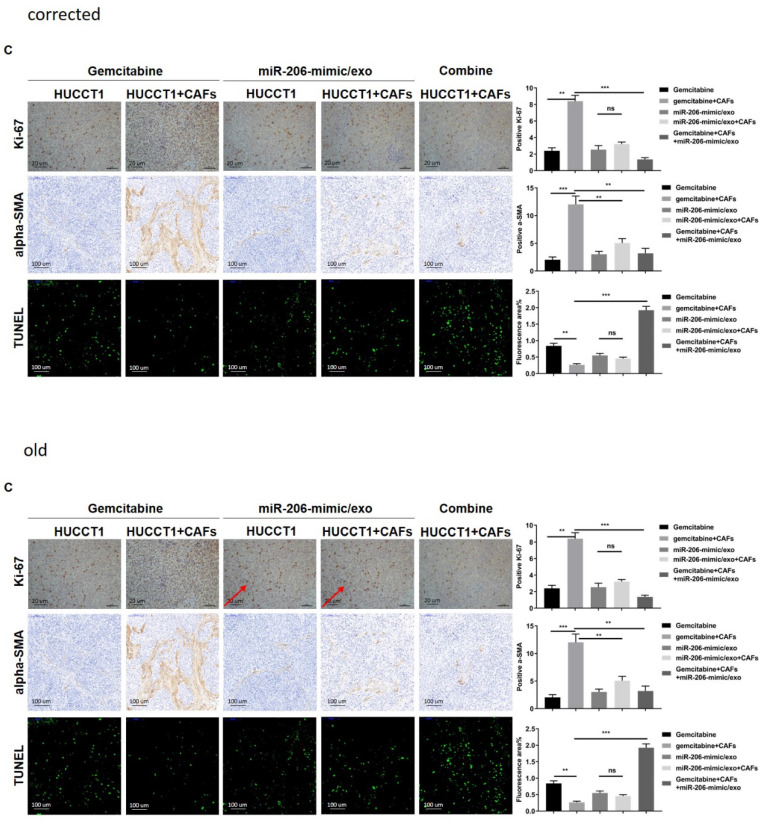
** C.** Corrected figure.

