# Supplementary material for: MiR-206 suppresses the deterioration of intrahepatic cholangiocarcinoma and promotes sensitivity to chemotherapy by inhibiting interactions with stromal CAFs
Source: Int J Biol Sci. 2022 Jan 1;18(1):43–64. doi: 10.7150/ijbs.62602 (PMC8692143; doi:10.7150/ijbs.62602)
Supplement: Supplementary file 1 — Supplementary figures and table. [file ijbsv18p0043s1.pdf]

Supplemental table and figures

Table S1 Primers used in this study

Gene	Forward (sequence 5'-3')	Reverse
miR-206	GCCCGCTGGAATGTAAGGAAGT	CCAGTGCAGGGTCCGAGGT
alpha-SMA	AAAAGACAGCTACGTGGGTGA	GCCATGTTCTATCGGGTACTTC
E-cadherin	CGAGAGCTACACGTTCACGG	GGGTGTCGAGGGAAAAATAGG
Vimentin	GACGCCATCAACACCGAGTT	CTTTGTCGTTGGTTAGCTGGT
IL-1beta	ATGATGGCTTATTACAGTGGCAA	GTCGGAGATTCGTAGCTGGA
IL-6	ACTCACCTCTTCAGAACGAATTG	CCATCTTTGGAAGGTTCAGGTTG
IL-8	TTTTGCCAAGGAGTGCTAAAGA	AACCCTCTGCACCCAGTTTTC
VEGF-alpha	ATGAACTTTCTGCTCTCTTGGGTACA	GCAGATGTGACAAGCCAAGGCGGTG
CXCL12	ATTCTCAACACTCCAAACTGTGC	ACTTTAGCTTCGGGTCAATGC
TGF-beta1	GGCCAGATCCTGTCCAAGC	GTGGGTTTCCACCATTAGCAC
Nanog	TTTGTGGGCCTGAAGAAAACT	AGGGCTGTCCTGAATAAGCAG
Sox2	GCCGAGTGGAAACTTTTGTCG	GGCAGCGTGTACTTATCCTTCT
Oct4	CTGGGTTGATCCTCGGACCT	CCATCGGAGTTGCTCTCCA
LASP1	TGCGGCAAGATCGTGTATCC	GCAGTAGGGCTTCTTCTCGTAG
Anxa2	TCTACTGTTCACGAAATCCTGTG	AGTATAGGCTTTGACAGACCCAT
U6	CCAGTGCAGGGTCCGAGGT	TGCGGGTGCTCGCTTCGCAGC
beta-actin	CATGTACGTTGCTATCCAGGC	CTCCTTAATGTCACGCACGAT

Figure S1

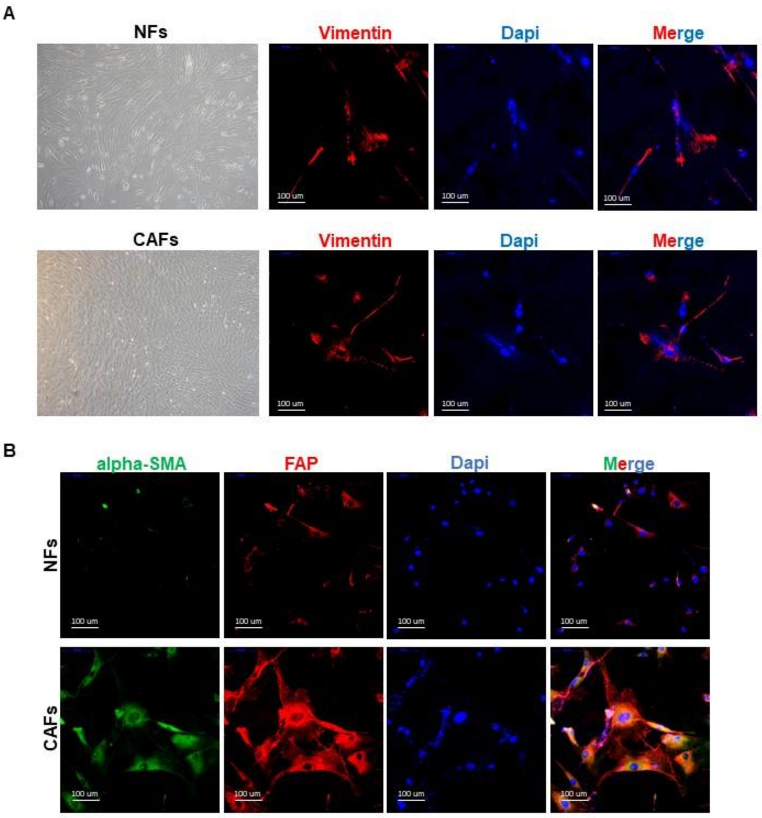

Isolation and identification of NFs and CAFs (A) Images of isolated NFs and CAFs and identification based on Vimentin expression. Scale bar=100 μm. (B) Expression of the activation markers alpha-SMA and FAP in paired NFs and CAFs was detected. Scale bar=100 μm.

Figure S2

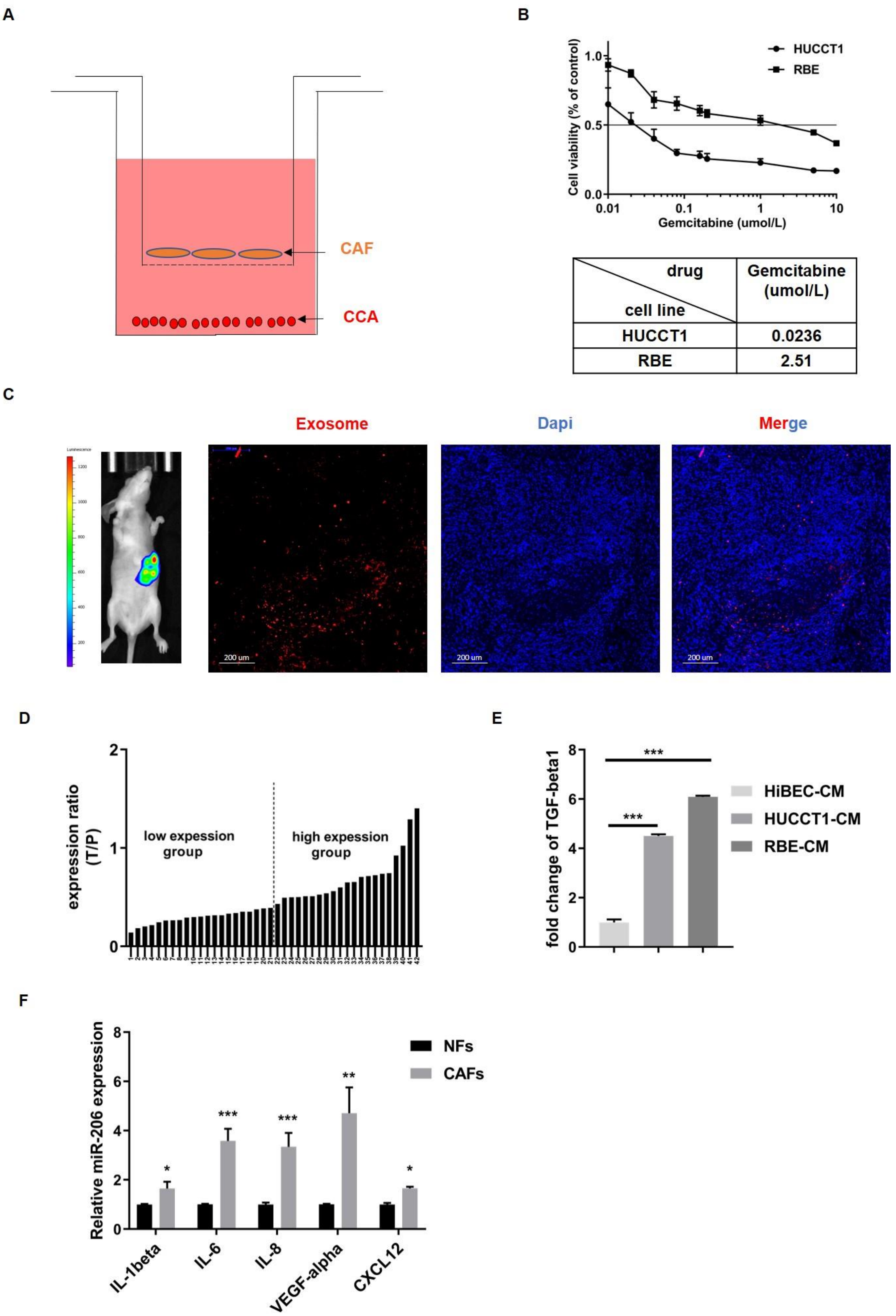

cell line \ drug	Gemcitabine (umol/L)
HUCCT1	0.0236
RBE	2.51

(A) CCA cells and CAFs were co-cultured in this study. CAFs and CCA cells were seeded into the upper or lower Transwell chamber of a 6-well plate. (B) The IC50 value of gemcitabine in the HUCCT1 and RBE cell lines was assessed and calculated. (C) An orthotopic mouse model was established, and photographs are shown in the left panel. Then, the mice were injected with

exosomes via the tail vein. Liver tissues were harvested for sectioning 2 days later. Exosomes were labelled with red fluorescence, and the cell nuclei were labelled with blue fluorescence (right panel). Scale bar=200 μm. (D) The ratio of the miR-206 levels in iCCA tissues compared to those in adjacent normal tissues was calculated and sorted. Forty-two patients were divided into two groups, with the median miR-206 expression level as the cut-off, and these groups were named the low and high expression groups. (E) relative concentration of TGF-beta1 in CM of HiBEC, HUCCT1 and RBE cell lines was detected and analyzed. (F) relative concentration of IL-1beta, IL-6, IL-8, VEGF-alpha and CXCL12 in CMs of HiBEC, HUCCT1 and RBE cell lines was detected and analyzed. *P <0.05, **P <0.01, ***P<0.001

Figure S3

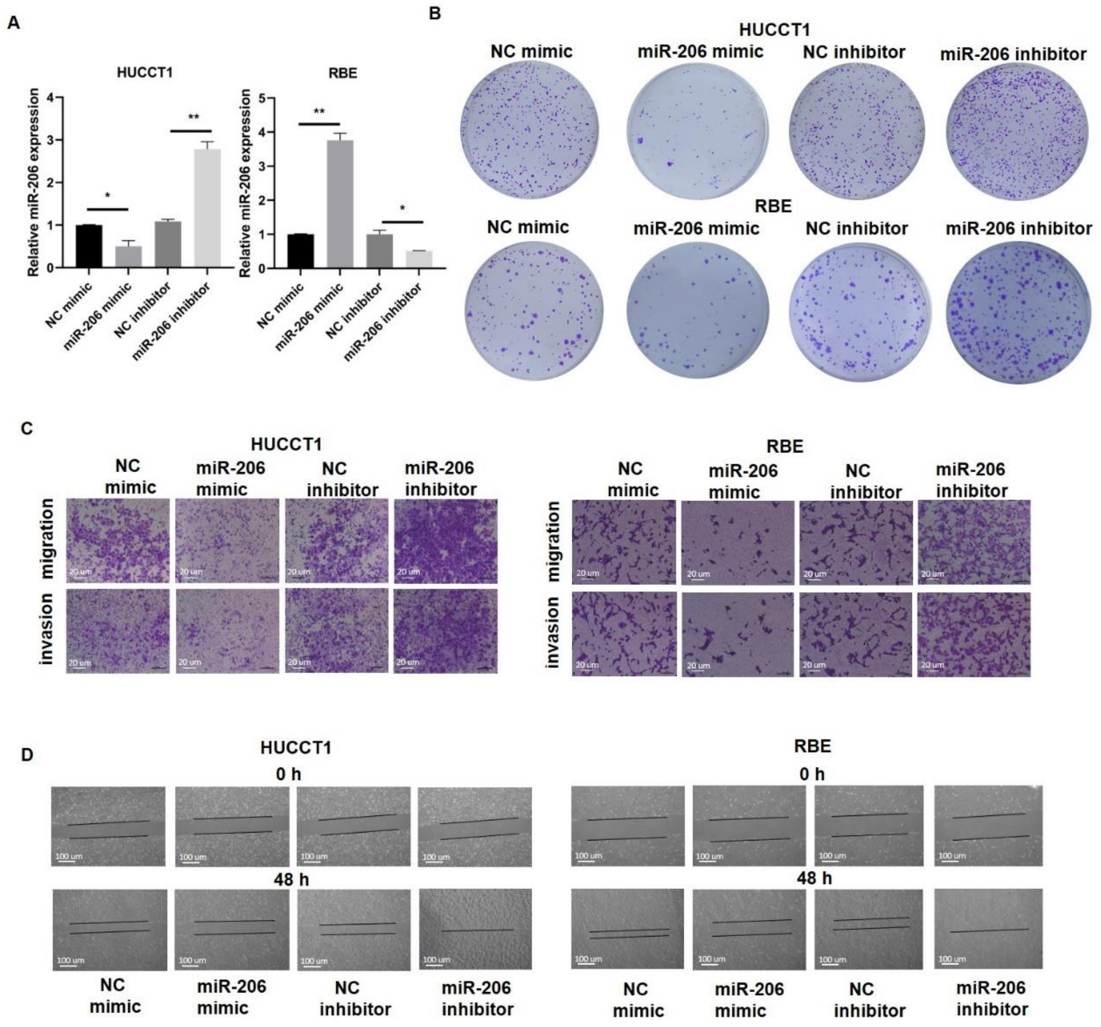

(A) Effects of the miR-206 mimic and inhibitor were confirmed. (B) Representative images of colony formation by HUCCT1 and RBE cells transfected with the miR-206-mimic or miR-206-inhibitor. (C) Representative images of Trans-well migration and invasion assays. Scale bar= 20 μm. (D) Representative images of wound healing assays. Scale bar= 100 μm

Figure S4

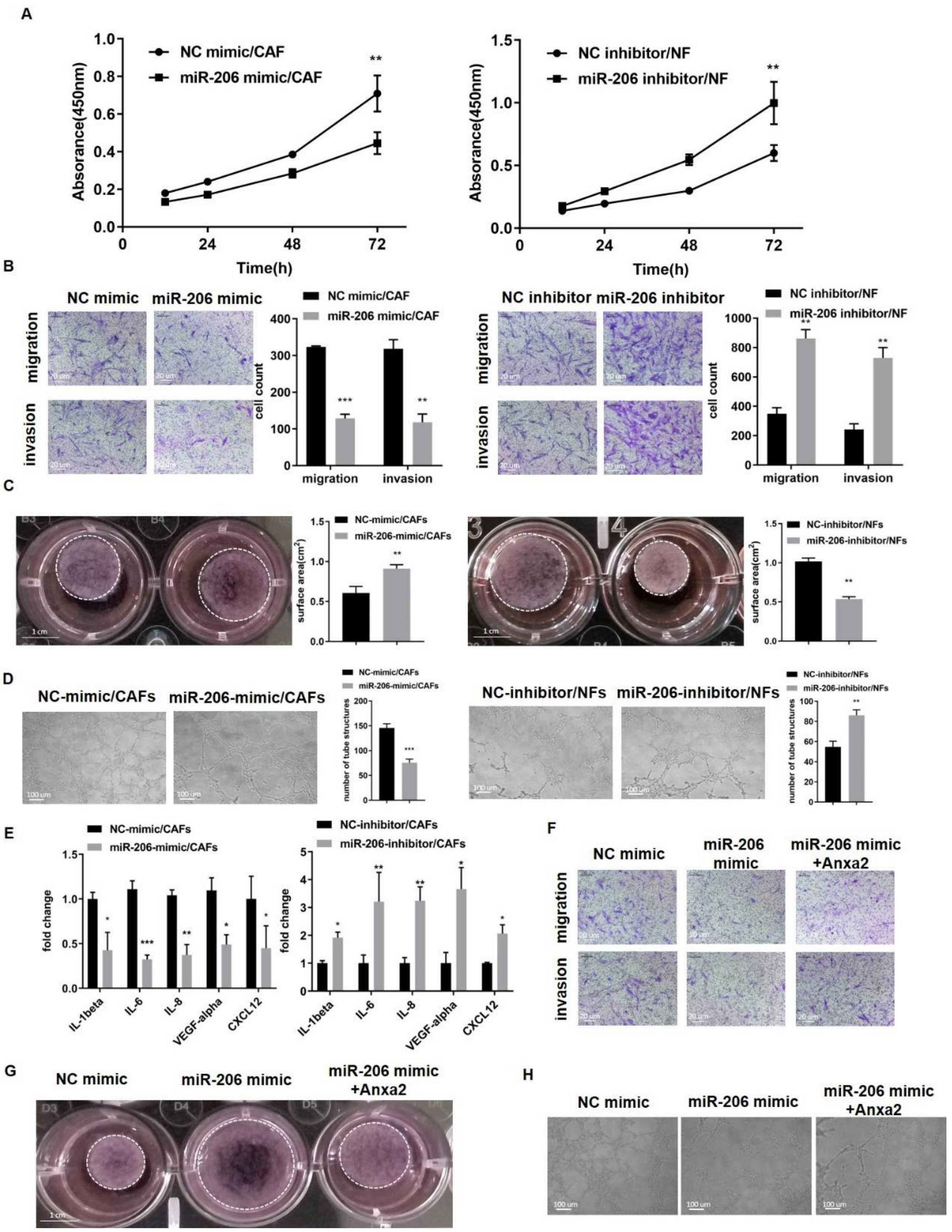

To study the effects of miR-206 on fibroblast biological behaviors, including proliferation, migration, collagen contraction promotion, angiogenesis and secretion, miR-206 expression was increased in CAFs and inhibited in NFs. (A-E) CCK-8, Transwell migration (scale bar = 20 μm), collagen contraction (scale bar = 1cm), vascular formation (scale bar = 100 μm) and ELISA assays were carried out. Representative images are presented. (F-H) The role of Anxa2 in the miR-206-mediated regulation of CAF activity was studied. Representative images of Transwell migration,

collagen contraction and vascular formation assays are shown. *P <0.05, ** P <0.01, *** P <0.001

Figure S5

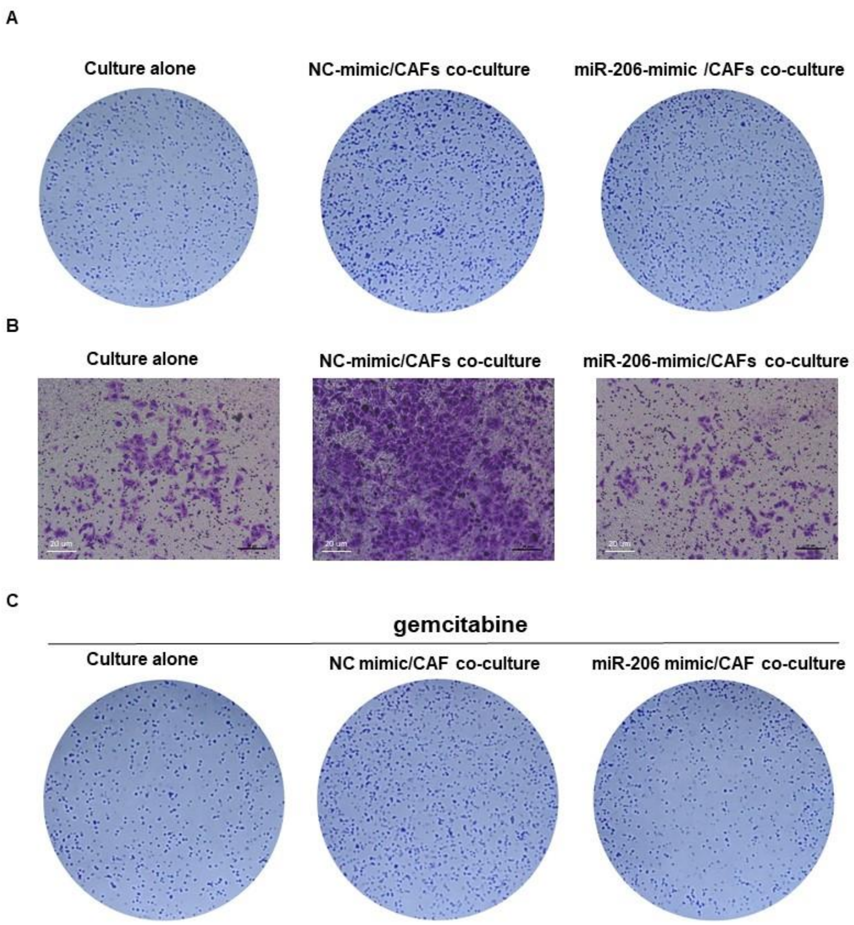

(A-B) Representative images of HUCCT1 cell colony formation and migration (scale bar = 20 μm) when cultured alone, cocultured with NC-mimic/CAFs and cocultured with miR-206-mimic/CAFs are presented. (C) Representative images of HUCCT1 cell colony formation after treatment with gemcitabine.

Figure S6

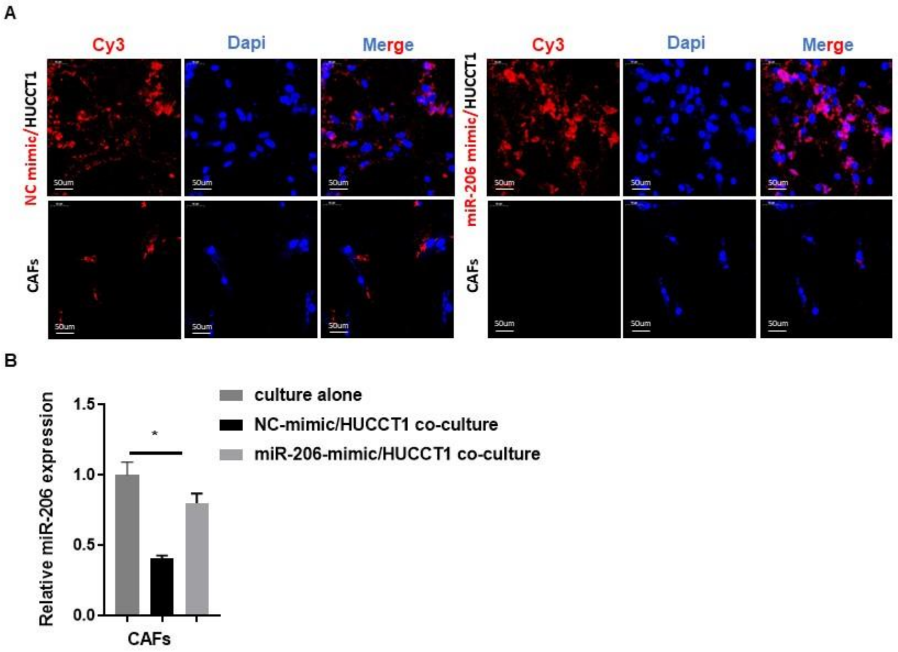

(A) MiR-206-mimic or NC mimic was labelled with Cy3 and transfected into HUCCT1 cells. After coculturing these cells with CAFs for 48 hours, the labelled miR-206 were not observed in the CAFs, and representative images were captured. Scale bar=50 μm. (B) The level of miR-206 in the co-cultured CAFs was analyzed by qPCR. **P* <0.05, ***P* <0.01, ****P* <0.001

Figure S7

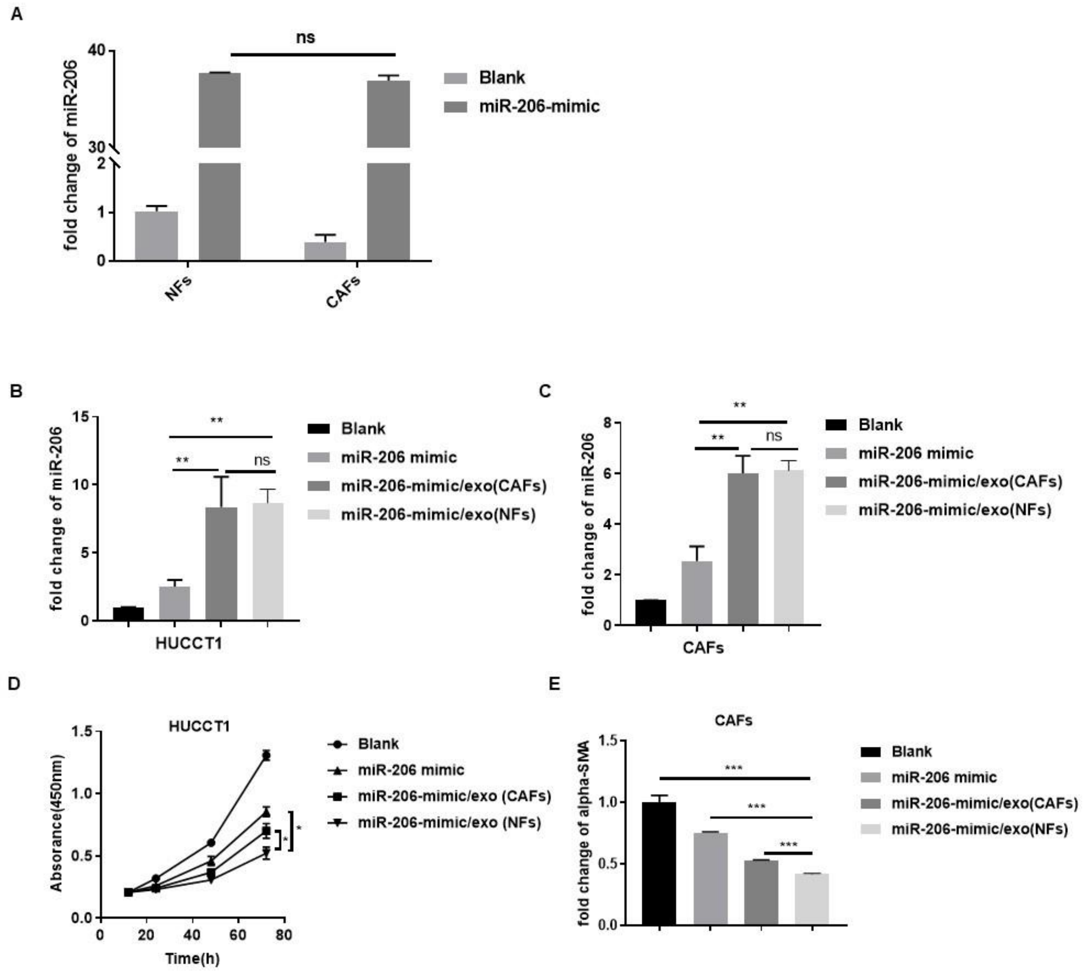

(A) Effect of miR-206 transfection into NF- and CAF-derived exosomes was confirmed by qPCR. No significant difference in the miR-206 levels was observed between the transfected NF- and CAF-derived exosomes. (B-C) HUCCT1 cells and CAFs were treated with the free miR-206 mimic, miR-206-mimic/exo (CAFs) or miR-206-mimic/exo (NFs). The miR-206 levels in recipient HUCCT1 cells and CAFs were detected, and the cells treated with modified exosomes exhibited higher levels

of miR-206 than those treated with the free miR-206 mimic. (D) CCK-8 assays revealed that the miR-206-mimic/exo (NFs) limited HUCCT1 cell proliferation to a lower degree than the free miR-206 mimic and miR-206-mimic/exo (CAFs). (E) Moreover, alpha-SMA expression in CAFs was analyzed by qPCR, and the results showed that miR-206-mimic/exo (NFs) led to a more significant reduction in alpha-SMA expression in CAFs compared to the free miR-206 mimic and miR-206-mimic/exo (CAFs). **P* <0.05, ** *P* <0.01, *** *P* <0.001
